# Combinatorial treatment with polyI:C and anti-IL6 enhances apoptosis and suppresses metastasis of lung cancer cells

**DOI:** 10.18632/oncotarget.15862

**Published:** 2017-03-02

**Authors:** Wai Hoe Lau, Xiphias Ge Zhu, Shamaine Wei Ting Ho, Shu Chun Chang, Jeak Ling Ding

**Affiliations:** ^1^ Department of Biological Sciences, Faculty of Science, National University of Singapore, Singapore 117543, Singapore; ^2^ Taipei Medical University, College for Medical Science and Technology, Taipei 110, Taiwan

**Keywords:** lung cancer cells, polyI:C-TLR3 suppression of survival and metastasis, anti-IL6 antibody, JAK2/STAT3 antagonists, cytokines and caspase 3/7 apoptosis

## Abstract

Activation of TLR3 stimulates cancer cell apoptosis and triggers secretion of inflammatory cytokines. PolyI:C, a TLR3 agonist, activates immune cells and regresses metastatic lung cancer *in vivo*. Although polyI:C reportedly kills lung carcinomas, the mechanism remains elusive. Here, we demonstrated that polyI:C suppressed the proliferation and survival of metastatic (NCI-H358 and NCI-H292) and non-metastatic (A549) lung cancer cells. Notably, A549, NCI-H292 and NCI-H358 which are inducible by polyI:C, expressed low-to-medium level of TLR3 protein, and were susceptible to polyI:C treatment. By contrast, NCI-H1299, which endogenously expresses high level of TLR3 protein, was insensitive to polyI:C. We showed that polyI:C stimulated pro-inflammatory cytokines associated with survival and metastasis in a cell type-specific manner. While A549 and NCI-H292 released high levels of IL6, IL8 and GRO, the NCI-H358 cells endogenously secretes abundant levels of these cytokines, and was not further induced by polyI:C. Thus, NCI-H358 was resistant to the inhibition of cytokine-dependent metastasis. NCI-H1299, which was unresponsive to polyI:C, did not produce any of the pro-inflammatory cytokines. Treatment of A549 with a combination of polyI:C and anti-IL6 antibody significantly decreased IL6 production, and enhanced polyI:C-mediated killing and suppression of oncogenicity and metastasis. While polyI:C stimulated the phosphorylation of STAT3 and JAK2, blockade of these proteins enhanced polyI:C-mediated suppression of survival and metastasis. Taken together, polyI:C alone provoked apoptosis of lung cancer cells that express low-to-medium levels of functional TLR3 protein. The combinatorial treatment with polyI:C and anti-IL6 enhanced polyI:C-mediated anticancer activities through IL6/JAK2/STAT3 signalling, and apoptosis via TLR3-mediated caspase 3/8 pathway.

## INTRODUCTION

Non-small cell lung cancer (NSCLC) is the most common type of lung carcinoma with poor prognosis [1]. Over 90% of the deaths of cancer patients is caused by metastasis, which is formed by the spread of disseminated primary tumor cells to distant anatomic sites [2]. In view of its high incidence and mortality rate, an effective modality to control lung cancer metastasis is urgently needed.

Toll-like receptors (TLRs) are a family of type I transmembrane proteins [3], best known to activate immune response against pathogens by recognizing conserved pathogen-associated molecular patterns expressed on microbes, and endogenous danger-associated molecular patterns released from stressed or dying cells [4, 5]. Upon engagement of its ligand, a TLR signals via activation of NF-κB [6] and interferon regulatory factors (IRF3/7) [7], to promote the expression of cytokines, chemokines and matrix metalloproteinases (MMPs), all of which initiate inflammatory response [8].

In healthy tissues, TLRs participate in the onset of innate immune defense but they are activated under pathological conditions, leading to chronic inflammation which creates a favourable environment for tumor initiation and progression [9]. Overexpression of TLRs purportedly promotes oncogenic transformation and metastatic potential [10]. However, the mechanistic role of TLRs in cancer is controversial. Tumor cells aberrantly express an array of TLRs [11], whereupon activation, may perform dual opposing functions, either to enhance host anti-tumor immunity or promote cancer survival [12, 13]. For example, the TLR4 ligand (LPS) causes resistance to anticancer therapies of ovarian and lung cancers [12–14] whereas activation of TLR9 by CpG DNA causes apoptosis of lung cancer cells [17]. TLR3, which is aberrantly expressed in hepatocellular carcinoma (HCC) [14], breast [15], melanoma [16], and metastatic lung carcinoma [17], was recently reported to trigger apoptosis in these cancers, when induced. The expression of TLR3 in HCC tissues reportedly exerts a synergistic effect on apoptosis and inhibition of cell proliferation, MMP-2 expression, and angiogenesis [14, 18]. In mice models of breast and lung cancers, TLR3 activation was reported to: (a) elicit chemoattraction of cytotoxic lymphocytes to the growing tumor [15, 19, 20], (b) induce secretion of type I interferon (IFN) and inflammatory cytokine/chemokine and (c) enhance anti-tumor immune responsiveness [16, 21].

PolyI:C, a TLR3 agonist, which activates immune cells to fight cancer progression, was deemed a potent adjuvant for treating melanoma [22], metastatic lung [17] and cervical cancer [23]. PolyI:C induces secretion of interferon (IFN)-β and provokes natural killer or cytotoxic T cells to activate anti-tumor immunity [24]. On the other hand, polyI:C also directly arrests tumor growth and induces apoptosis of human cancer cells [15, 25]. The expression of TLR3 is variable amongst lung cancer cells, prompting us to question the capacity of TLR3 to activate apoptosis and how TLR3 ligands on their own or in combination with other drugs may be administered to treat lung cancer. Hiltonol, a drug analogue of polyI:C has been under phase II clinical trial as an adjuvant therapy against melanoma, prostate, and breast cancer [26, 27]. However, variable efficacies of Hiltonol warrants further investigation on its molecular mechanisms of action. Here, we used polyI:C as a model ligand to delineate the differential outcomes of TLR3-mediated anti-cancer effects. Hitherto, information is lacking on the functional effects of polyI:C on TLR3 in lung cancer. We examined four NSCLC cell lines - the non-metastatic A549 and NCI-H1299 and the metastatic NCI-H292 and NCI-H358. We demonstrated heterogeneity of TLR3 protein levels in the subsets of lung cancer cell lines associated with differential susceptibility to polyI:C. To date, the correlation between TLR3 protein level and the susceptibility of the cancer cells to polyI:C is unknown. Here, we observed that the primary prerequisite for the activation of TLR3-mediated apoptosis pathway is (i) low-to-medium levels of TLR3 protein in the cancer cell lines and (ii) sufficient engagement of polyI:C and TLR3 for activation of TLR3 signalling, where cell death ensues regardless of the aggressiveness of the cancer cell type. Notably, polyI:C stimulates pro-inflammatory cytokines associated with survival and metastasis in a cell type-specific manner. We demonstrated that polyI:C alone significantly suppressed the survival, oncogenicity and metastasis of A549, NCI-H292 and NCI-H358 cells via TLR3-mediated caspase 3/8 apoptosis pathway. Furthermore, the combinatorial treatment with polyI:C and anti-IL6 antibody enhanced polyI:C-suppression of survival, oncogenicity and metastasis of A549 cells through both IL6/JAK2/STAT3 signalling and TLR3-mediated caspase 3/8 apoptosis pathways. Thus, we have uncovered the association of pro-inflammatory cytokine expression profiles with the TLR3-mediated apoptosis pathway, which could be useful therapeutic indicators for the prediction of susceptibility to polyI:C treatment with good prognosis. Our data supports that polyI:C (its analogue, Hiltonol) is a promising adjuvant in combination with anti-IL6 antibody and/or JAK2/STAT3 inhibitors, for lung cancer immunotherapy.

## RESULTS

### PolyI:C induced TLR3 expression in only some cancer cells

**Figure 1:**
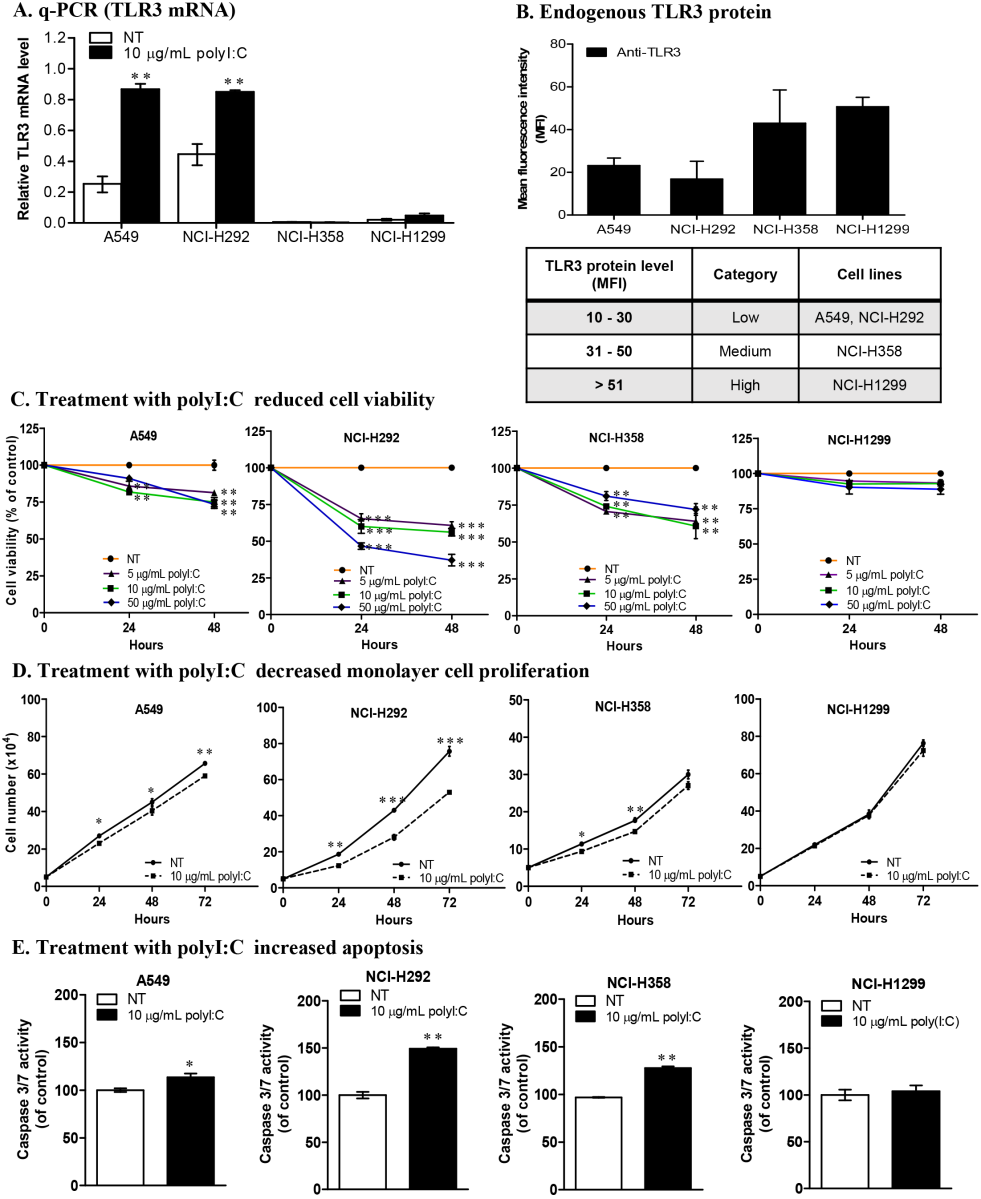
Lung cancer cells exhibit differential TLR3 expression and susceptibility to polyI:C **(A)** Real-time qPCR analysis of *Tlr3* mRNA levels relative to *Hprt*, in lung cancer cells after 24-h polyI:C treatment. **(B)** Flow cytometric analysis of endogenous TLR3 protein levels presented as mean fluorescence intensity (MFI) of TLR3 protein labelled with FITC-conjugated anti-TLR3 antibody after normalization with IgG isotype control. Table shows the range of TLR3 protein levels and the category of lung cancer cells. **(C)** Cell viability of lung cancer cells treated with increasing doses of polyI:C (5, 10, 50 μg/mL) over 48 h. NT indicates non-treated PBS control (without polyI:C). **(D)** Monolayer cell proliferation was measured by total cell number. Cells were treated with 10 μg/mL polyI:C over 72 h. **(E)** Apoptosis measured by activation of caspase 3/7 activity, was analysed by caspase-glo 3/7 luminescent assay. Cells were treated with 10 μg/mL polyI:C for 24 h followed by 30 min incubation with caspase-glo 3/7 reagent. The caspase 3/7 activity is presented as percent of luminescence of polyI:C-treated cells relative to that of control NT cells. *P<0.05, **P<0.01, ***P<0.001.

Constitutive expression of endogenous TLR3 in cancer cells could be an indicator of susceptibility or resistance to polyI:C stimulation. Hence, we examined the TLR3 mRNA levels in lung, liver, breast and colon cancer cells. We found variations of TLR3 mRNA expression amongst the cancer types tested (Supplementary Figure 1A), and heterogeneity was also observed within the different cell lines of the lung and hepatocellular cancers (HCC). Notably, lung cancer cell lines, A549 (non-metastatic) and NCI-H292 (metastatic), express substantial endogenous levels of TLR3 mRNA when not treated with polyI:C (Figure 1A, NT white bars) but they express low levels of TLR3 protein (Figure 1B and Supplementary Figure 1B) compared to those of NCI-H358 and NCI-H1299. The TLR3 protein levels in A549, NCI-H292 and NCI-H358 were low-to-medium. Flow cytometry showed TLR3 to be intracellularly expressed in lung cancer cells, unlike the aberrant expression of cell surface TLR3 in HCC [15]. PolyI:C stimulation significantly increased TLR3 expression in A549 and NCI-H292 (Figure 1A, Supplementary Figure 1A), which prompted us to further study the impact of differential engagement of polyI:C with TLR3 on the different lung cancer cell lines. The heterogeneity of the endogenous and polyI:C-induced levels of TLR3 protein in the lung cancer cell lines provide us with good models to investigate the differential impact of polyI:C on survival and metastatic potential as a means to understand why Hiltonol (a drug analogue of polyI:C) elicited differential therapeutic efficacy in cancer patients.

### PolyI:C induced variable cell death to different cancer cell types

The susceptibility of cancer cells to polyI:C was assessed using MTT cell viability assay. Dose response curve of polyI:C showed that 24-h treatment with 10 μg/mL achieved IC50 for NCI-H292, and 20% maximum killing of A549 and NCI-H358 (Supplementary Figure 1C). Treatment of the lung, liver and colon cancer cell lines with actinomycin D (Supplementary Figure 1D), a known anti-neoplastic agent for transcription inhibition, showed significant decrease in cell viability up to 50% at 24-h treatment, indicating that these cell lines are highly sensitive to a cytotoxic agent. Since polyI:C is known to be an anti-cancer agent, albeit, with variable efficacy, we next examined the levels of susceptibility of different cancer cell lines to polyI:C. The effects were found to be variable amongst lung (Figure 1C), colon (Supplementary Figure 1E), and liver (Supplementary Figure 1F) cancer cells.

### Lung cancer cells displayed differential susceptibility to polyI:C treatment

PolyI:C stimulated variable cell death in different lung cancer cell lines, with significant decrease in the viability of A549, NCI-H292 and NCI-H358 in a time-dependent manner, but NCI-H1299 remained unaffected (Figure 1C). After 48-h treatment, NCI-H292 showed more cell death dose-dependently of polyI:C, with 40% and 52% reduction at 10 and 50 μg/mL polyI:C, respectively. We observed that after 48-h, 10 μg/mL polyI:C reduced the viability of A549 and NCI-H358 by 25% and 39%, respectively, but higher doses of polyI:C did not cause further cell death. Intriguingly, NCI-H292, which expresses low level of TLR3 protein, was significantly killed by polyI:C treatment. However, NCI-H1299, which expresses high level of TLR3 protein, remained unperturbed by polyI:C. Hence, polyI:C treatment seemed to have an inverse impact on the viability of lung cancer cells depending on the TLR3 protein level. Henceforth lung cancer cells which express low-to-medium levels of TLR3 protein, are referred to as ‘polyI:C-susceptible’, and 10 μg/mL polyI:C was applied for all subsequent studies unless otherwise stated.

### PolyI:C inhibited the proliferation and survival of polyI:C-susceptible lung cancer cells

It is known that dsRNA is a potent inducer of type I interferons (IFNs) [28], exerting pro-apoptotic and anti-proliferative effects on neoplastic cells [29, 30]. To determine whether reduction of cell viability resulted from inhibition of cell division or induction of cancer cell death, we examined the impact of 10 μg/mL polyI:C on the monolayer cell proliferation of the four lung cancer cell lines: A549, NCI-H292, NCI-H358, and NCI-H1299, for 24, 48 and 72 h. We showed that after 48-h treatment, polyI:C significantly reduced the NCI-H292 and NCI-H358 monolayer cell number (1.7-fold) while A549 appeared less affected (1.4-fold decrease) (Figure 1D). On the other hand, NCI-H1299, which expresses high endogenous level of TLR3 protein, was not induced by polyI:C treatment. Consistently, polyI:C decreased the doubling time of A549, NCI-H292 and H358, but not NCI-H1299. Taken together, these results suggest that engagement of TLR3 by polyI:C significantly inhibited cell proliferation by decreasing the doubling time of A549, NCI-H292, and NCI-H358 (Supplementary Figure 1G). TLR3 activation may regress cancers by inducing apoptosis and inhibiting cell proliferation. After 24-h treatment, we observed significant increase in apoptosis of NCI-H292, with 50% increase of caspase 3/7 activity compared to untreated controls. A549 and NCI-H358 appeared less affected, with only 13% and 28% increase in caspase 3/7 activity, respectively. Again, apoptosis of NCI-H1299 was not induced by polyI:C (Figure 1E). By labelling the nuclei of apoptotic cells with caspase 3/7 green fluorescence, we found 2-fold increase in the number of caspase 3/7–positive cells in NCI-H292 and NCI-H358, but only 1.4-fold increase in A549, consistently indicating that polyI:C directly induced apoptosis of these polyI:C-susceptible cell lines (Supplementary Figure 2A).

### PolyI:C attenuated the oncogenicity of susceptible lung cancer cells

**Figure 2:**
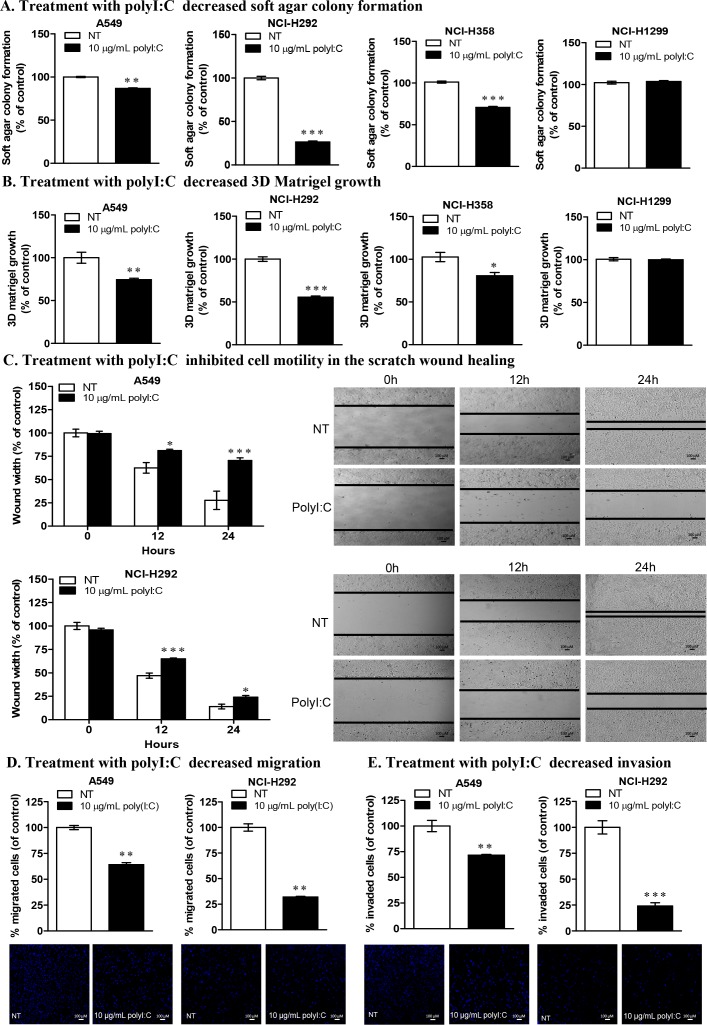
PolyI:C suppresses oncogenicity, cellular motility, migration and invasion of A549 and NCI-H292 cells **(A)** Soft colony formation of lung cancer cells treated with polyI:C for assessment of anchorage-independent growth over 12 days. Cells were treated with 10 μg/mL polyI:C consecutively every 3 days over a period of 12 days and the viability of the colony formed in the soft agar was measured by Alamar blue assay. Control NT cells were treated with PBS (without polyI:C). Enumerated soft agar colonies are presented as percent polyI:C-treated cells relative to NT cells. **(B)** 3D matrigel growth of cells treated with 10 μg/mL polyI:C consecutively every 3 days over 7 days. The viability of the cell colony formed in the matrigel was quantified and data presented as described in **(A)**. **(C)** Scratch wound healing assay to measure cellular motility and wound closure rate of A549 and NCI-H292 cells treated with polyI:C at different time intervals (0, 12, 24 h). The wound closure rate and wound width restoration was analysed using ImageJ software. The wound width is presented as percent polyI:C-treated cells relative to NT cells. The representative images of the wounded areas were taken after polyI:C treatment at different time intervals. **(D** and **E)** Migration and invasion of A549 and NCI-H292 were determined by transwell migration /invasion assays. Cells were treated with 10 μg/mL polyI:C for 24 h and the migrated /invaded cells underneath the transwell insert were stained by Hoechst 33342, and counted under fluorescence microscopy. The invasion potency was determined using 2% matrigel pre-coated transwell invasion assay. The data are presented as percent of polyI:C-treated cells relative to NT. Images were taken at 40x magnification. Bar, 100 μM; *P<0.05, **P<0.01, ***P<0.001.

We next used soft agar colony formation and 3D matrigel growth to investigate anchorage-independent growth in the soft agar and three-dimensional culture growth in the matrigel (which represents a reconstituted basement membrane), respectively. These assays evaluate the capacity of polyI:C to attenuate the oncogenicity, tumorigenicity and malignant transformation of the polyI:C-susceptible lung cancer cells [31, 32]. After treating the cell lines with 10 μg/mL polyI:C for 12 days, we observed significant attenuation of anchorage-independent growth of A549, NCI-H292 and NCI-H358 by 21%, 74% and 29%, respectively, as indicated by decreased cell number and colony size, formed in the soft agar. Again, NCI-H1299 was insensitive to polyI:C treatment (Figure 2A). These results suggest that polyI:C induces anoikis in A549, NCI-H292, and NCI-H358 cells by attenuating their anchorage-independent growth. Treatment with 10 μg/mL polyI:C for 7 days significantly attenuated the three-dimensional growth of A549, NCI-H292 and NCI-H358 by 26%, 44% and 21%, respectively in matrigel, suggesting that polyI:C would presumably elicit a significant impact on suppression of oncogenic transformation of lung cancer cells in the laminin- and collagen- rich basement membrane *in vivo*. Consistently, NCI-H1299 was insensitive to polyI:C treatment (Figure 2B). Thus polyI:C treatment suppresses the oncogenic transformation of A549, NCI-H292 and NCI-H358, but not NCI-H1299.

### PolyI:C suppressed the metastatic potential of susceptible lung cancer cells

Cancer cells acquire migratory and invasive characteristics to promote metastasis [33]. PolyI:C was reported to suppress cellular migration of human HCC cells [34] and neuroblastoma cells [35]. Here, by performing scratch wound healing, transwell migration and invasion assays, we showed the capacity of polyI:C to suppress migration and invasion of A549 and NCI-H292. Scratch wound healing assay showed that polyI:C decreased the motility of A549 and NCI-H292 by 42% and 68%, respectively, with a significant inhibition of wound-width restoration within 24-h time course (Figure 2C). Additionally, IncuCyte live cell imaging showed that the motility of polyI:C-treated A549 and NCI-H292 were significantly slowed down compared to the untreated controls (Supplementary Movie 1), supporting that polyI:C inhibited wound closure rate and wound-width of A549 and NCI-H292 in a dose-dependent manner over the 24-h time course. Concordantly, polyI:C treatment of NCI-H292 significantly suppressed its migration by 68% and invasion by 72% and polyI:C treatment of A549 suppressed its migration by 36% and invasion by 28% (Figure 2D, 2E). However, NCI-H358 appeared relatively unresponsive, with no impact on its cellular motility, migration and invasion (Supplementary Figure 2B, 2C, 2D). These results indicate that polyI:C exerts anti-metastatic properties, which suppress the metastatic potential of A549 and NCI-H292 cells (low TLR3), but not of NCI-H358 (medium TLR3). These observations prompted us to examine the specificity of TLR3 signalling and the implication of polyI:C-induced pro-/anti-inflammatory cytokines in these cell lines. Henceforth, we focussed our attention on A549 and NCI-H292, unless otherwise stated (for comparisons).

### TLR3 neutralization and knockdown diminished the impact of polyI:C-killing of lung cancer cells

**Figure 3:**
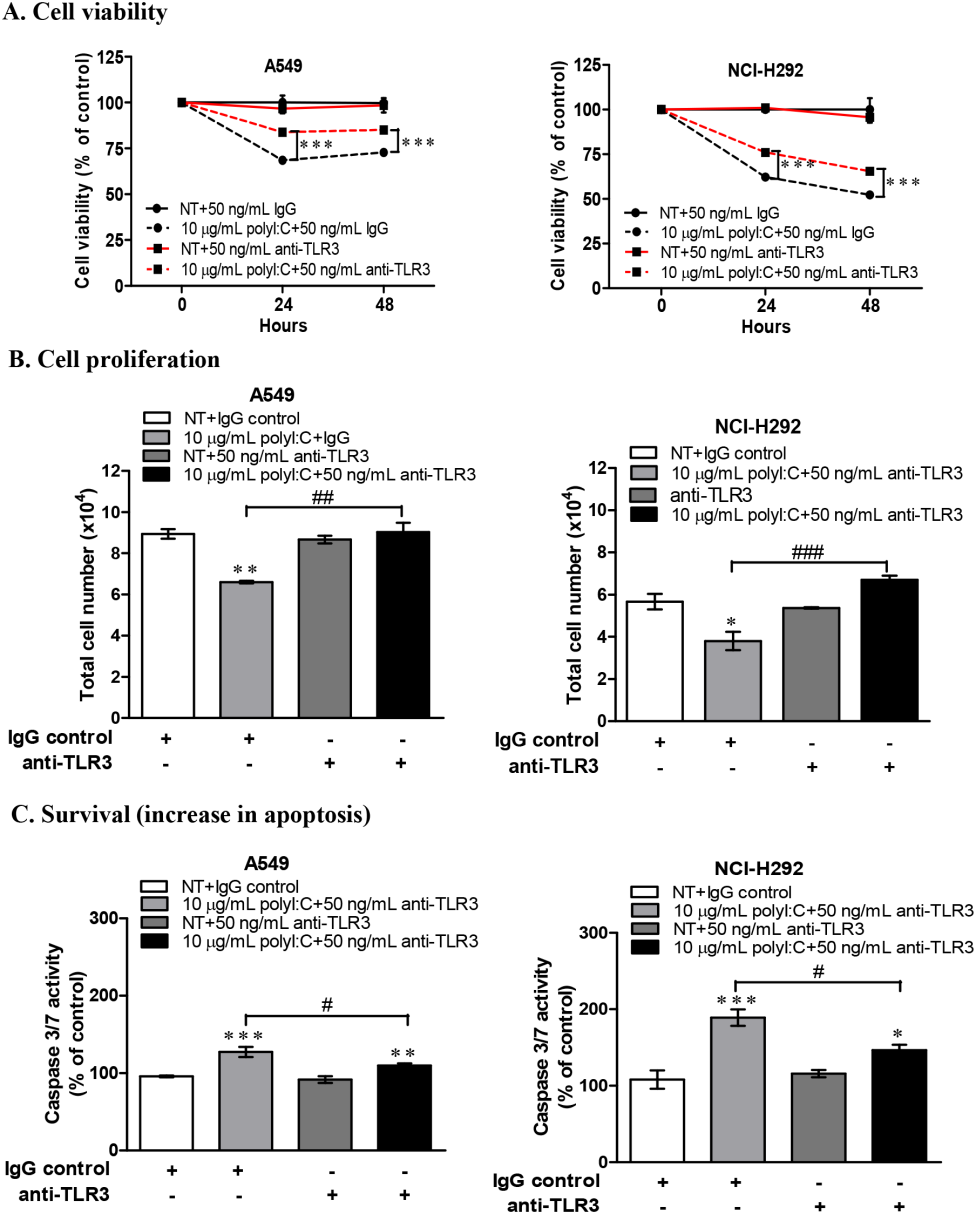
TLR3 neutralization decreased polyI:C-suppression of cell viability, monolayer cell proliferation and survival of A549 and NCI-H292 cells Cells were incubated with purified anti-human TLR3 (Functional grade, e-Bioscience) for 1 h and then treated with 10 μg/mL polyI:C for 24 and 48 h. Control cells were pre-incubated with IgG isotype control and then treated with PBS (without polyI:C). **(A)** Cell viability was analyzed by MTT assay and data are presented as percent of polyI:C-treated cells to the cells pre-incubated with IgG isotype control or anti-TLR3 relative to control cells. **(B)** Monolayer cell proliferation assay was performed after cells were treated as described above. Monolayer cell proliferation data were determined and presented as above. **(C)** Apoptosis was measured via activation of caspase 3/7 activity. Same treatment conditions were followed as above. The caspase 3/7 activity was analysed by caspase-glo 3/7 assay. Apoptosis was calculated and data presented as described above. *P<0.05, **P<0.01, ***P<0.001. *P<0.05, **P<0.01, ***P<0.001 indicate polyI:C treated cells compared to NT cells. ^#^P<0.05, ^##^P<0.01, ^###^P<0.001 indicate cells with TLR3 neutralization compared to IgG isotype control.

To confirm that TLR3 is directly involved in the polyI:C-mediated growth inhibition and apoptosis, we blocked TLR3 with a neutralizing monoclonal antibody to disrupt TLR3-mediated signalling in A549 and NCI-H292 cells. Since we utilized these two susceptible lung cancer cell lines to investigate the underlying mechanisms of polyI:C-mediated killing, it was rational to examine the loss-of-function of TLR3 in these cell lines to validate the specificity of polyI:C on the activation of TLR3 signalling. These cells were pre-treated with TLR3 neutralizing antibody for 1 h prior to treatment with polyI:C for 24 h. TLR3 antibody blocked TLR3 signalling without decreasing the viability of the lung cancer cells. TLR3 antibody significantly reversed the polyI:C-mediated suppression of the cell viability (Figure 3A), cell proliferation (Figure 3B) and survival (Figure 3C) in A549 and NCI-H292 within 24 h. These results suggest that TLR3 antibody specifically blocked TLR3 in A549 and NCI-H292 (which express low level of TLR3 protein) to impair the binding of polyI:C with TLR3 and therefore disrupted TLR3 signalling, and resulted in the attenuation of polyI:C-mediated cancer killing.

**Figure 4:**
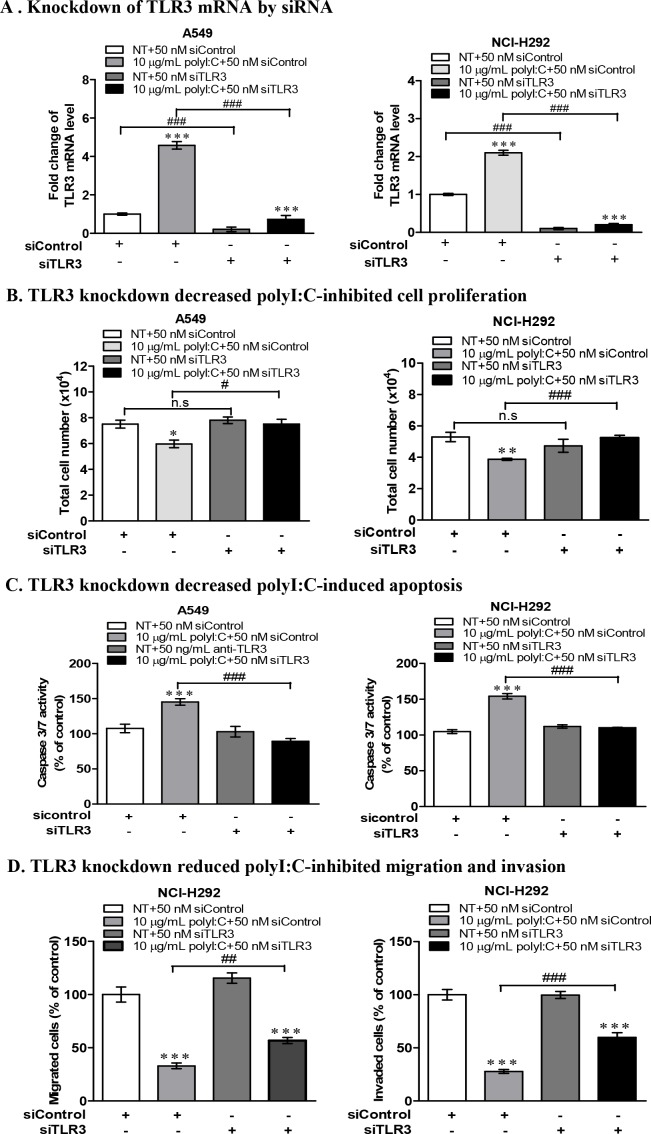
TLR3 knockdown by siRNA decreased polyI:C-suppression of monolayer cell proliferation, survival, migration and invasion of A549 and NCI-H292 **(A)** TLR3 mRNA level of A549 and NCI-H292 after knockdown with 50 nM TLR3-siRNA (siTLR3) for 24 h, compared to scrambled RNA control (siControl). After knockdown, the cells were treated with 10 μg/ml polyI:C for another 24 h. Data are presented as *Tlr3* mRNA level relative to a housekeeping gene (*Hprt*) compared to NT control (not treated with polyI:C). **(B)** Monolayer cell proliferation assay (of total cell numbers) shows that TLR3 knockdown decreased the polyI:C-killing of A549 and NCI-H292. **(C)** TLR3 knockdown cells were treated as described above and caspase 3/7 activity was measured by caspase-glo 3/7 assay, showing decreased the polyI:C-mediated apoptosis. **(D)** TLR3 knockdown NCI-H292 cells were treated as above, and the cell migration and invasion were assayed, showing decreased the polyI:C-mediated suppression. The migrated and invaded cells which appeared underneath the transwell insert were stained with Hoechst 33342 and counted by fluorescent microscopy. For all of the above functional assays, the results are presented as percent of polyI:C-treated cells with siControl or siTLR3 relative to NT. *P<0.05, **P<0.01, ***P<0.001 indicate polyI:C treated cells vs. NT. n.s, non-significant; ^#^P<0.05, ^##^P<0.01, ^###^P<0.001 indicate cells with TLR3 knockdown vs. scrambled control siRNA.

To further validate the role of TLR3 signalling in polyI:C-suppression of cell growth /apoptosis, A549 and NCI-H292 cells were subjected to TLR3 siRNA knockdown (Figure 4A), which reduced the impact of polyI:C on the cellular behaviours. The polyI:C-inhibition of cell proliferation and apoptosis were reduced (Figure 4B, 4C). TLR3 knockdown also diminished the polyI:C-suppression of migration and invasion of NCI-H292 (Figure 4D). These results suggest that TLR3 knockdown reduced the effect of polyI:C since there was no TLR3 protein for polyI:C to engage with. There was substantial loss of polyI:C-mediated suppression of survival and metastatic potential in both A549 and NCI-H292 cells. Together, these results support that polyI:C functions as a pro-apoptotic, anti-proliferative and anti-metastatic agent, which specifically targets TLR3 signaling pathway in susceptible lung cancer cells.

### PolyI:C stimulated differential secretion of pro-/anti- inflammatory cytokines

**Figure 5:**
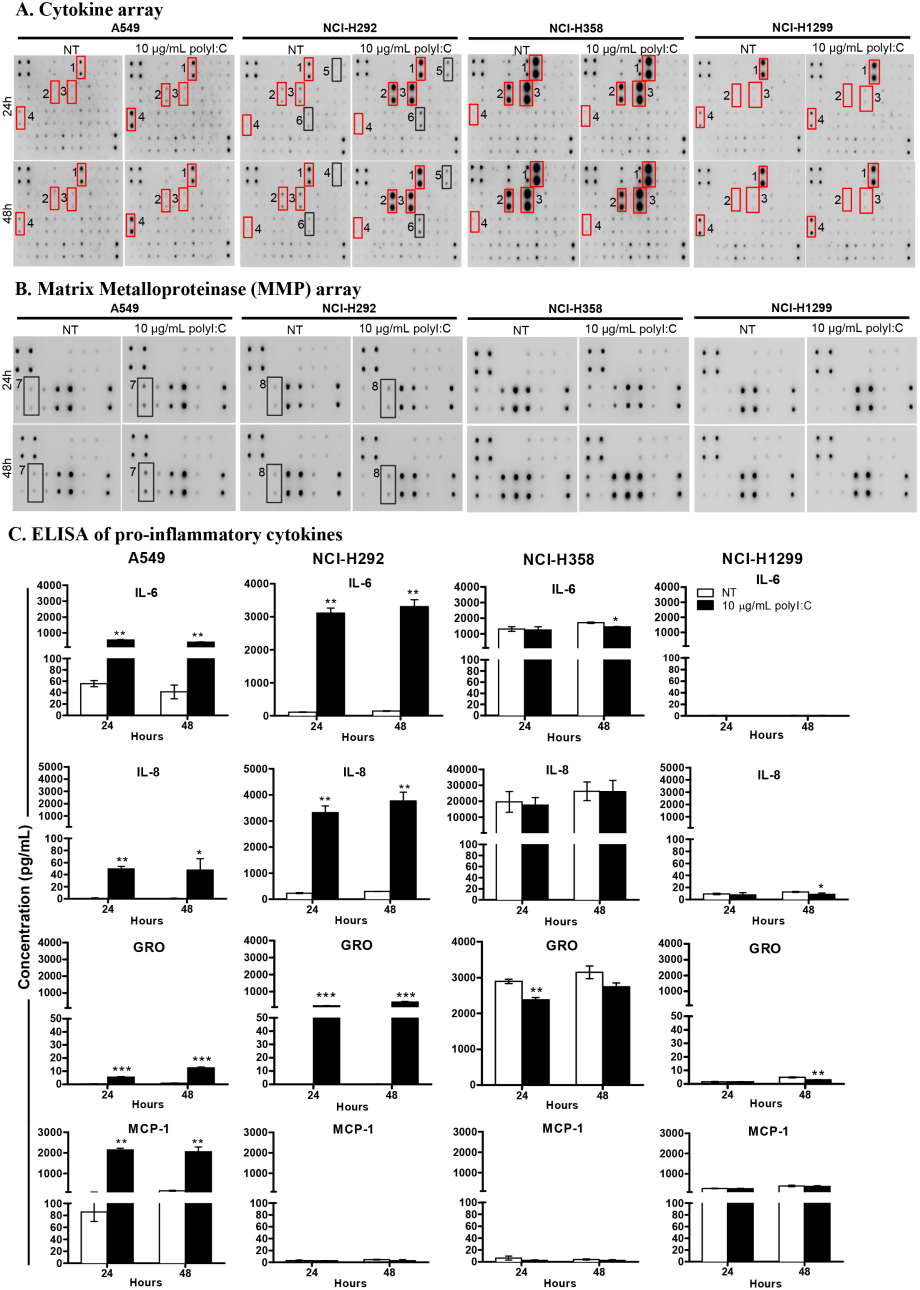
PolyI:C stimulated differential secretion of pro-/anti- inflammatory cytokines and matrix metalloproteinase in lung cancer cells Cells were treated with 10 μg/mL polyI:C for 24 and 48 h and the culture supernatants were collected for measurement of secreted cytokines using human cytokine array and ELISA. **(A)** A549 secreted high levels of GRO, IL6, IL8 and MCP-1; NCI-H292 generated high levels of GRO, IL6, IL8, IL-1α, and RANTES but produced low level of MCP-1; NCI-H358 secreted saturating levels of GRO, IL6, IL8 but produced low level of MCP-1; NCI-H1299 secreted very low level of GRO, IL6, IL8 and moderate level of MCP-1. Red boxes indicate significant increase of cytokines secreted by polyI:C-treated cells compared to NT cells and these cytokines were then validated by ELISA. **(B)** PolyI:C stimulated differential secretion of matrix metalloproteinase (MMP) in lung cancer cells. Cells were treated as described above and the culture supernatants were measured for MMP using human MMP cytokine array. Different levels of MMP isoforms were produced: A549 secreted MMP10 while NCI-H292 secreted MMP13, whereas NCI-H359 and NCI-H1299 were unresponsive to polyI:C and did not secrete any MMPs. The numbers next to the red/black boxes indicate: (1) GRO, (2) IL6, (3) IL8, (4) MCP-1, (5) IL-1α, (6) RANTES, (7) MMP10, (8) MMP13. **(C)** ELISA confirmed differential quantities of IL6, IL8, GRO and MCP-1 secreted from different lung cancer cells. *P<0.05, **P<0.01, ***P<0.001.

The results obtained thus far prompted us to investigate whether, in addition to cancer cell killing and suppression of metastatic potential, the functional consequences of polyI:C are associated with the production of pro-/anti- inflammatory cytokines. Thus, we examined the effects of polyI:C on pro-/anti-inflammatory cytokine secretion, which are associated with survival and metastasis of the lung cancer cells. The profile of pro-/anti- inflammatory cytokines and matrix metalloproteinase secreted (MMP) from A549, NCI-H292, NCI-H358, and NCI-H1299 cells were determined by cytokine and MMP arrays. Figure 5A, 5B, Supplementary Figure 3A show that polyI:C stimulated the secretion of pro-inflammatory cytokines in a cell-type specific manner. The A549 was most highly responsive, with significant secretion of IL-6, IL-8, GRO and MCP-1. The NCI-H292 showed increase in IL-1α, IL-8, GRO and RANTES but decrease in MCP-1. The A549 and NCI-H292 secreted substantial amounts of MMP10 and MMP13, respectively (Figure 5B and Supplementary Figure 3B). Conversely, NCI-H1299 did not secrete any of the cytokines tested, and NCI-H358 was found to constitutively synthesize high endogenous levels of IL-6, IL-8 and CXCL9, even without polyI:C stimulation, suggesting that polyI:C did not have an impact on the production of these pro-inflammatory cytokines in NCI-H358 and NCI-H1299 (Figure 5A, 5B). Notably, NCI-H1299 which already expresses high endogenous levels of TLR3 protein (see Figure 1B), was apparently resistant/non-responsive to polyI:C stimulation. On the other hand, NCI-H358 which expresses a medium level of TLR3 and releases abundant endogenous levels of these cytokines, did not produce more cytokines when stimulated with polyI:C, probably because the cytokines were already at saturation levels. This may also explain why NCI-H358 resisted suppression of the cytokine-dependent metastatic potential (motility, migration and invasion), when treated with polyI:C.

Several candidate pro-/anti- inflammatory cytokines secreted from the lung cancer cells lines (A549, NCI-H292, NCI-358, and NCI-H1299) were compared with those of HCC cell lines (SNU499, Huh1, HuH7, HepG2) and colon (WiDr) cancer cells, after 24- and 48-h polyI:C treatment. In agreement with the cytokine array results, quantification of the candidate cytokines by ELISA showed that polyI:C increased secretion of IL-6, IL-8, and GRO from A549 and NCI-H292 cells in a time-dependent manner (Figure 5C). We observed that A549 but not NCI-H292, produced high amounts of MCP-1. However, in contrast to A549 and NCI-H292, the endogenous and polyI:C-induced IL-6, IL-8, and GRO were abundantly secreted by NCI-H358 and scarcely by NCI-H1299. Nevertheless, polyI:C did not induce TNFα, IL-1β, IL10 and IL-12p40 in HCC and colon cancer cell lines tested (Supplementary Figure 3B). Thus, the polyI:C-induced secretion of these pro-/anti- inflammatory cytokines was apparently specific to lung cancer cells but not to HCC and colon cancer cells.

### PolyI:C treatment of A549 and NCI-H292 activated caspase 3, STAT3 and JAK2

**Figure 6:**
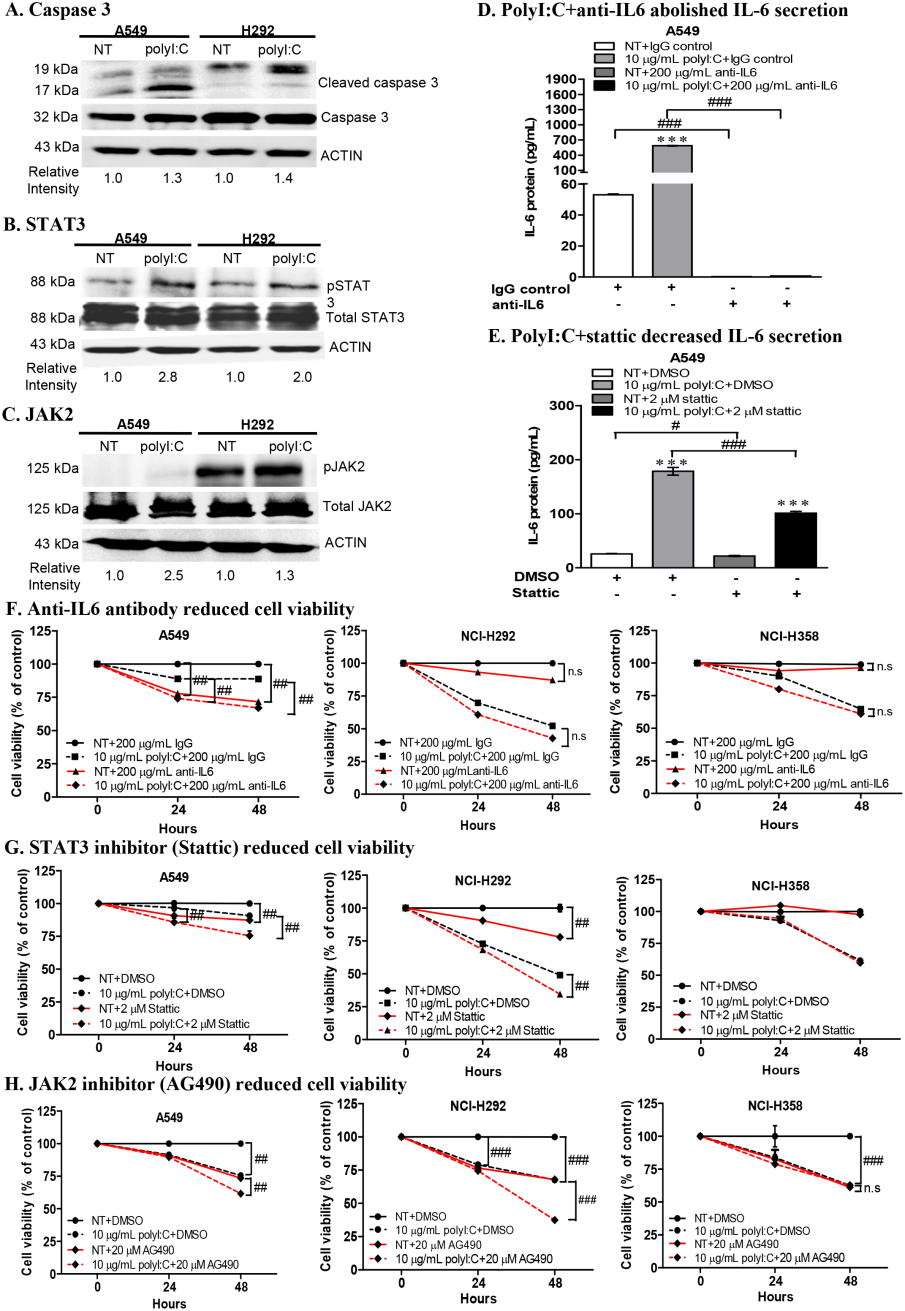
PolyI:C increased caspase 3, STAT3, JAK2 activities and combinatorial treatment with polyI:C+anti-IL6 or polyI:C+inhibitors (Stattic /AG490) enhanced the polyI:C-killing of A549 and NCI-H292 **(A)** Treatment of cells with 10 μg/mL polyI:C for 24 h increased the expression of cleaved caspase 3 (19, 17 kDa). Whole cell lysates were analysed by Western blot. PolyI:C stimulation increased phosphorylation of **(B)** STAT3 and **(C)** JAK2 in both A549 and NCI-H292. Band intensities were quantified by ImageJ software with β-actin as the loading control. The relative intensities were calculated by polyI:C-treated against control NT. **(D)** ELISA of cell culture supernatant of A549 after combinatorial treatment with polyI:C+anti-IL6 for 24 h shows abolishment of IL6 secretion. **(E)** ELISA of cell culture supernatant of A549 after combinatorial treatment with polyI:C+Stattic for 24 h shows decreased IL6 secretion (black bars). **(F)** A combinatorial treatment with polyI:C+anti-IL6 antibody for 24 h enhanced polyI:C-killing of A549 but have no effect on NCI-H292 and NCI-H358. Cell viability was analyzed by MTT assay. Control cells (NT) were treated with IgG as isotype control and without polyI:C. Cell viability is presented as percent of polyI:C-treated cells +/− anti-IL6 antibody relative to NT. Combinatorial treatments with: **(G)** polyI:C+Stattic and **(H)** polyI:C+AG490 for 24 h enhanced polyI:C-killing of both A549 and NCI-H292 but have no effect on NCI-H358. Control cells (NT) were treated with DMSO as vehicle control and without polyI:C. Cell viability is presented as percent of polyI:C-treated cells +/− Stattic (G) or AG490 (H), relative to NT. *P<0.05, **P<0.01, ***P<0.001 indicate polyI:C treated cells vs. untreated cells. n.s, non-significant; ^#^P<0.05, ^##^P<0.01, ^###^P<0.001 indicate cells treated with a combinatorial vs. no combinatorial treatment.

PolyI:C was reported to induce STAT3 phosphorylation in immortalized human bronchial epithelial cells, and pharmacological inhibitors of STAT3 and JAK2 essentially abolished polyI:C-induced STAT3 activation [36]. Thus we further investigated the direct signalling pathway linking TLR3 activation to STAT3 phosphorylation (which enhances IL-6 secretion) in A549 and NCI-H292. We demonstrated that polyI:C increased the: (i) expression of activated/cleaved caspase 3 (19, 17 kDa) (Figure 6A), (ii) phosphorylation of STAT3 at tyrosine 705 (pY705) (Figure 6B) and (iii) phosphorylation of JAK2 (Figure 6C), in both A549 and NCI-H292 cells. The endogenous caspase 3 (32 kDa), total STAT3 and JAK2 were not activated by polyI:C treatment. But cleaved caspase 3, and phosphorylated STAT3 and JAK2 were not significantly increased in both NCI-H358 and NCI-H1299 (Supplementary Figure 3C). Additionally, neutralization of secreted IL6 with anti-IL-6 antibody abolished both the basal and polyI:C-induced IL-6 secretion in A549 (Figure 6D). Inhibition of STAT3 activity by Stattic partially decreased (1.2-fold) the basal level of IL-6 and significantly decreased (1.8 fold) the polyI:C-induced IL-6 secretion by A549, as compared to vehicle controls (Figure 6E), indicating that polyI:C induced STAT3 phosphorylation leading to enhancement of IL-6 secretion in A549.

### Impact of combinatorial treatment with polyI:C, anti-IL6 antibody, STAT3 and JAK2 antagonists

Since A549 is an aggressively growing, albeit non-metastatic cell type, and NCI-H292 & NCI-H358 are metastatic, and all three cell lines are responsive to polyI:C, we performed combinatorial treatment of these cell lines and compared their responses wherever appropriate. We found that the above combinatorial treatment resulted in:

### Abrogation of cell viability, proliferation and survival via JAK2-STAT3 signalling

Constitutive activation of JAK2 and STAT3 is known to enhance cell survival, proliferation, and metastasis in multiple cancers [37, 38]. Inhibition of STAT3 activity was reported to induce apoptosis in lung adenocarcinoma cells via IL6/JAK2/STAT3 signalling pathway [39]. These information prompted us to examine whether neutralization of IL6 with anti-IL6 antibody would enhance the anticancer effect of polyI:C on both A549 and NCI-H292, via TLR3-mediated IL6/JAK2/STAT3 activation. We observed that a combination of polyI:C and anti-IL6 antibody significantly increased polyI:C-killing of A549 cells by up to 22% after 48-h treatment, as compared to isotype antibody controls (Figure 6F; compare dashed black-red lines), indicating that neutralization of secreted IL6 by anti-IL6 antibody enhanced the anticancer effect of polyI:C. Additionally, IL6 antibody alone significantly decreased the viability of untreated A549, suggesting that anti-IL6 neutralized the basal level of IL6 and resulted in the suppression of A549 cell survival (Figure 6F; compare solid black-red lines). On the other hand, anti-IL6 did not elicit further impact on the polyI:C-killing of NCI-H292 and NCI-H358, although the NCI-H292 secretes a high level of polyI:C-induced IL6, suggesting that polyI:C killed NCI-H292 in an IL6-independent manner. Consistently, NCI-H358, which constitutively secretes abundant level of endogenous IL6 (even without polyI:C treatment, see Figure 5), appeared unperturbed by anti-IL6 antibody (Figure 6F).

Blockade of STAT3 activity by Stattic increased polyI:C-killing of the A549 and NCI-H292 cells by up to 15% after 48-h treatment with polyI:C, as compared to the vehicle controls (Figure 6G; compare dashed black-red lines). Furthermore, blockade of JAK2 activity by AG490 significantly increased the polyI:C-killing of A549 and NCI-H292, by up to 32% (Figure 6H; compare dashed black-red lines). Single treatment with Stattic or AG490 alone (without polyI:C) partially suppressed viability and survival of A549 and NCI-H292 (Figure 6G, 6H; solid black-red lines). For NCI-H358 cells, blockade of STAT3 and JAK2 did not activate polyI:C-mediated killing since neither STAT3 nor JAK2 was activated by polyI:C stimulation (Figure 6G, 6H). These results indicate that polyI:C specifically stimulates the TLR3-mediated activation of JAK2/STAT3 in A549 and NCI-H292.

**Figure 7:**
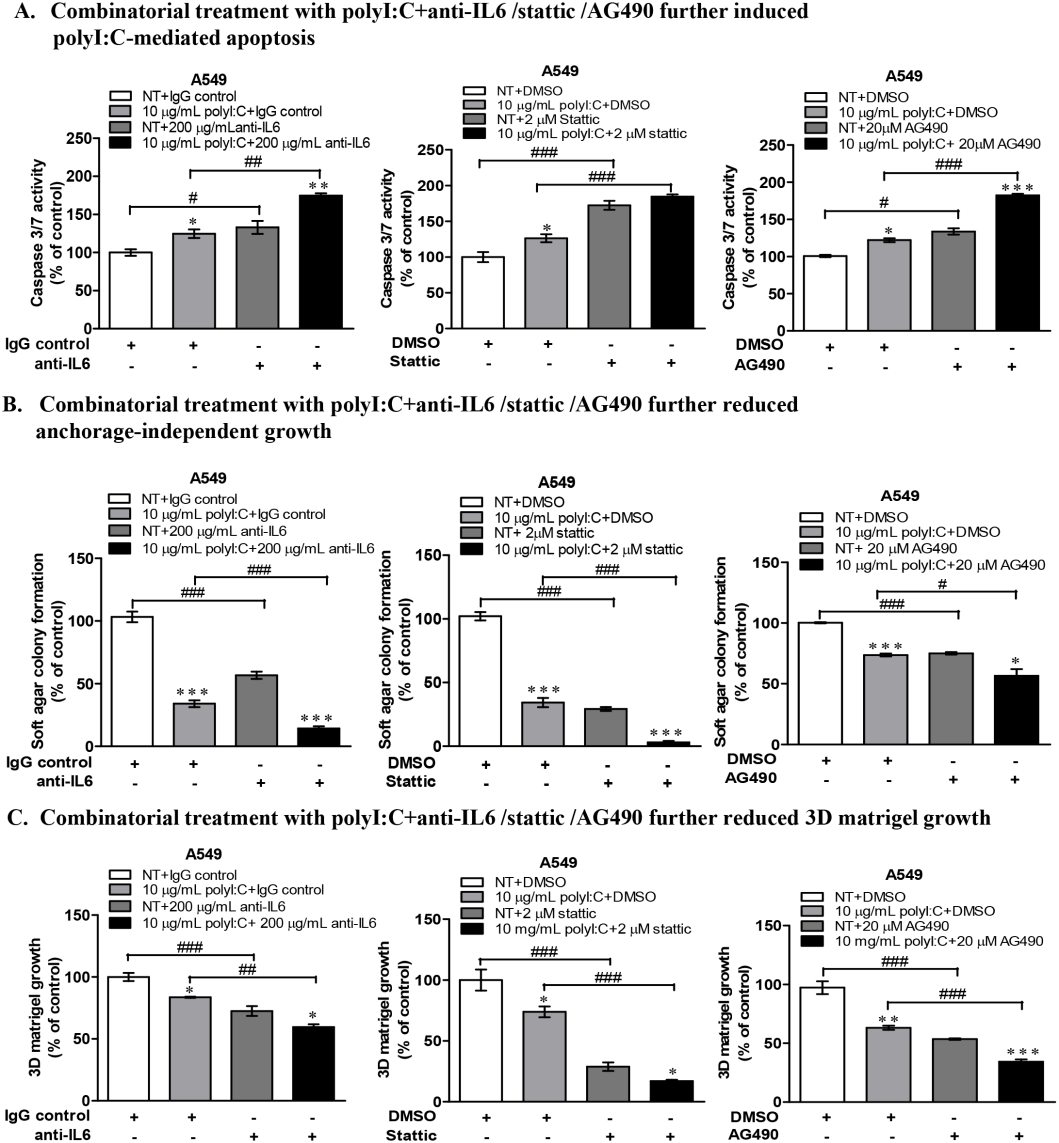
Combinatorial treatment of A549 cells with polyI:C+anti-IL6 or polyI:C+inhibitors (Stattic /AG490) enhanced polyI:C-killing and further reduced oncogenicity **(A)** Combinatorial treatments with anti-IL6 /Stattic /AG490 for 24 h enhanced polyI:C-induced apoptosis of A549. Caspase 3/7 activity was analyzed by caspase-glo 3/7 assay. Apoptosis is presented as percent of caspase 3/7 activity in the polyI:C treated cells +/− combinatorial treatment relative to NT. **(B)** Combinatorial treatments with anti-IL6 /Stattic /AG490 for 24 h further reduced anchorage-independent growth of A549. Cells were consecutively treated for 12 days and viability of the colony formed in the soft agar was analyzed by Alamar blue assay. Soft agar colony formation is presented as percent of polyI:C-treated cells +/− combinatorial treatment, relative to control cells. **(C)** Combinatorial treatments with anti-IL6 /Stattic /AG490 for 24 h further reduced 3D matrigel growth. Cells were consecutively treated for 7 days and viability of the colony formed in the matrigel was analyzed by Alamar blue assay.3D matrigel growth is presented as percent of polyI:C-treated cells +/− combinatorial treatment relative to NT. *P<0.05, **P<0.01, ***P<0.001 indicate polyI:C treated cells vs. untreated cells. n.s, non-significant; ^#^P<0.05, ^##^P<0.01, ^###^P<0.001 indicate cells under combinatorial vs. no combinatorial treatment.

Of significance is that A549 cells, an aggressively growing and proliferating lung cancer cell type, was strongly impacted by anti-IL6 antibody treatment, which substantially enhanced the polyI:C-induced apoptosis by increasing caspase 3/7 activity by up to 50% after 24-h treatment compared to isotype controls (Figure 7A). Similarly, blockade of STAT3 and JAK2 activities in A549 (Figure 7A) and NCI-H292 cells (Supplementary Figure 4A) significantly enhanced polyI:C-mediated apoptosis by increasing caspase 3/7 activity. Taken together, these data recapitulate polyI:C-killing through JAK2/STAT3 signalling via IL6-dependent apoptosis in A549, but IL6-independent apoptosis in NCI-H292.

### Abrogation of oncogenicity via disruption of JAK2-STAT3 signalling

We next investigated how disruption of IL6/JAK2/STAT3 signalling pathway, which enhanced polyI:C-killing of A549 and H292, might have a functional impact on their oncogenicity. We found that (a) combination of polyI:C and anti-IL6 antibody, (b) blockade of STAT3 by Stattic and (c) blockade of JAK2 by AG490, all significantly enhanced the ability of polyI:C to suppress anchorage-independent growth and three-dimensional culture growth in the matrigel, particularly with A549 cells (Figure 7B, 7C) and NCI-H292 cells (Supplementary Figure 4B, 4C). A combinatorial treatment with the STAT3 or JAK2 antagonists significantly decreased the number and size of the colonies formed, suggesting enhancement of the therapeutic efficacy of polyI:C where suppression of oncogenicity of A549 and NCI-H292 occurred through disruption of JAK2/STAT3 signaling. Taken together, these results indicate that single treatment with either anti-IL6 antibody or Stattic or AG490 is able to suppress the oncogenic transformation of A549 cells, but in combination, the suppression of oncogenicity of A549 and NCI-H292 was significantly enhanced.

### Abrogation of metastasis via disruption of JAK2-STAT3 signalling

**Figure 8:**
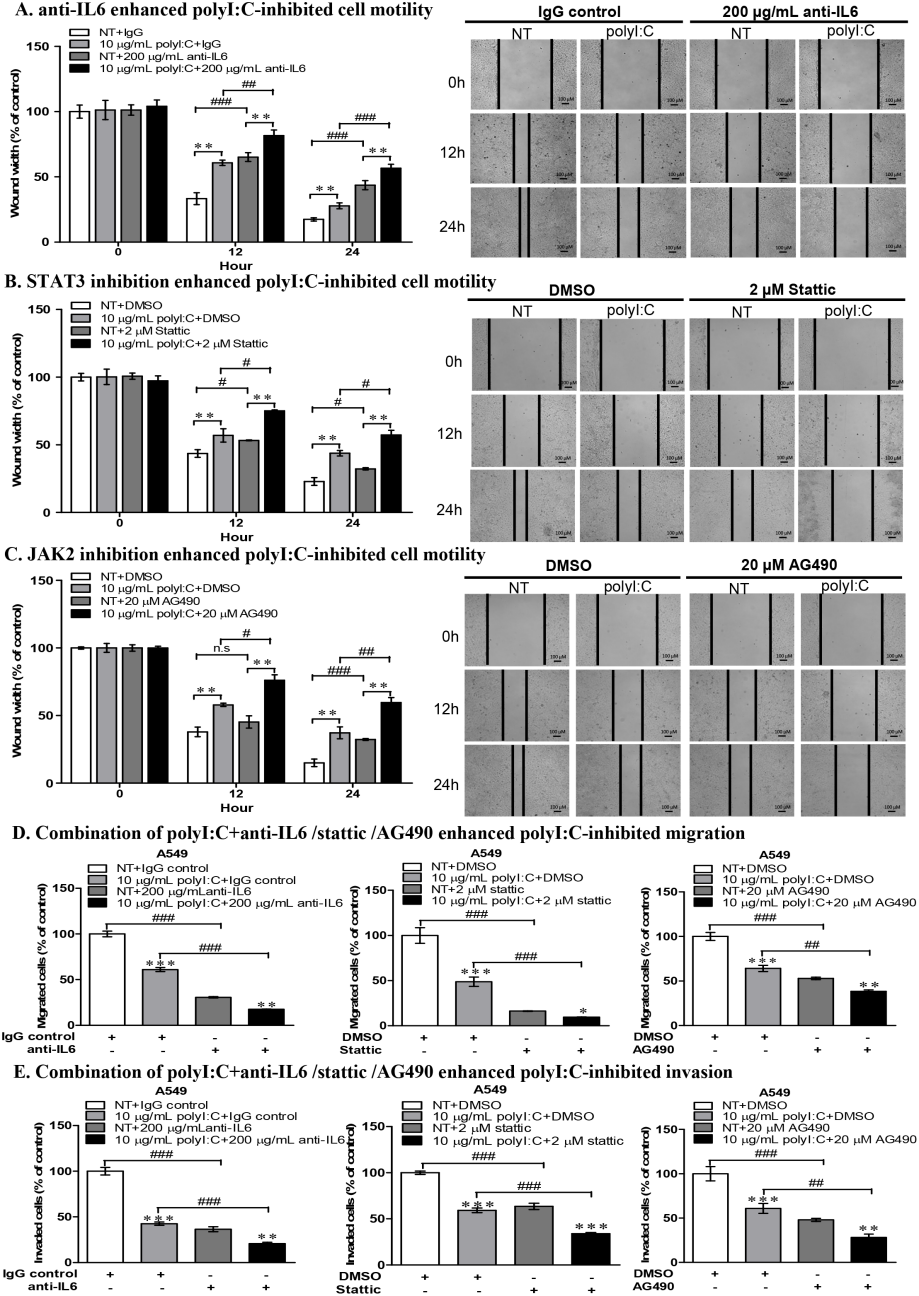
Combinatorial treatment of A549 with polyI:C+anti-IL6 or polyI:C+inhibitors (Stattic /AG490) enhanced polyI:C-killing and suppressed cell motility, migration and invasion Scratch wound assay to assess cellular motility and wound closure rate of A549 cells treated for different time intervals (0, 12, 24 h) with: **(A)** polyI:C+anti-IL6 antibody; **(B)** polyI:C+Stattic; **(C)** polyI:C+AG490. Wound closure rate and wound width were measured using ImageJ software. Wound width is presented as percent polyI:C-treated cells +/− combinatorial treatment relative to NT. The representative microscopy images of the wounded areas were examined under 40× magnification. **(D)** Migration and **(E)** Invasion were measured after 24-h treatment of A549 cells with polyI:C+anti-IL6 antibody or polyI:C+Stattic or polyI:C+AG490. The migrated or invaded cells underneath the transwell insert were stained by Hoechst 33342, and counted under fluorescence microscopy. The representative images of migrated cells were examined under ×40 magnification. The migrated and invaded cells are presented as percent of cells treated with a combinatorial treatment relative to control NT cells. Bar, 100 μM; *P<0.05, **P<0.01, ***P<0.001 indicate polyI:C-treated cells vs. untreated cells. n.s, non-significant; ^#^P<0.05, ^##^P<0.01, ^###^P<0.001 indicate cells treated with combinatorial vs. no combinatorial treatment.

In scratch wound healing assay, the combination of polyI:C and anti-IL6 antibody significantly enhanced polyI:C-inhibited wound restoration and cellular motility of A549 cells (Figure 8A). Blockade of STAT3 or JAK2 enhanced polyI:C-inhibited wound restoration and cellular motility in both A549 (Figure 8B, 8C) and NCI-H292 cells (Supplementary Figure 5A, 5B). Additionally, combination of polyI:C with anti-IL6 or stattic or AG490 inhibitors (which disrupt IL6/JAK2/STAT3 signalling), further significantly enhanced the polyI:C-inhibited migration and invasion of A549 (Figure 8D, 8E). Consistently, blockade of STAT3 and JAK2 activities in NCI-H292 cells also further significantly enhanced polyI:C-suppression of migration and invasion (Supplementary Figure 5C, 5D). These results support that polyI:C induces activation of JAK2 and STAT3, and that inhibition of JAK2 and STAT3 activities enhanced polyI:C-mediated killing, indicating that JAK2 and STAT3 are involved in survival and metastasis of A549 and NCI-H292 cells. Therefore, future studies may confirm the efficacy of cocktail treatment(s) including Hiltonol, anti-IL6 antibody and JAK2-STAT3 antagonists.

## DISCUSSION

The expression of TLR3 has been associated with the risk and prognosis of multiple cancers. Engagement of polyI:C with TLR3 induces apoptosis and subsequently activates infiltration of immune cells to destroy cancer cells [16, 40, 41]. Here, we have demonstrated that the endogenous levels of TLR3 mRNA are variable amongst lung, breast and colon cancer cell lines tested, and there is heterogeneity even amongst different cell lines within the same type of cancer, e.g. lung cancer (A549, NCI-H292, NCI-H358, NCI-H1299). We demonstrated that A549 (non-metastatic) and NCI-H292 (metastatic) constitutively express substantial level of TLR3 mRNA, and are inducible by polyI:C. However, NCI-H358 (metastatic) and NCI-H1299 (non-metastatic) seem to express low/basal levels of TLR3 mRNA, suggesting initially, that TLR3 level may not be linked to metastatic potential. Interestingly, despite the low levels of TLR3 mRNA in NCI-H358 and NCI-H1299 (Figure 1A), these cell lines constitutively express relatively higher levels of TLR3 protein (Figure 1B). This discordant relationship between TLR3 mRNA and TLR3 protein expression may be caused by a low stability of the TLR3 mRNA and/or post-translational modifications of the TLR3 protein. Therefore, the presence of TLR3 mRNA may not predict the expression of proportional level of functional TLR3 protein [42, 43]. This prompted us to investigate the underlying mechanisms of polyI:C-induced TLR3 activation, which led to differential suppression of the survival, oncogenicity and metastasis of these cell lines.

Activation of TLR3 by polyI:C in lung cancer cells triggers inflammation and inhibits growth through production of inflammatory cytokines, anti-proliferative proteins [44, 45] and upregulation of caspase-dependent apoptosis [46]. *In vivo* mice study showed that polyI:C regressed tumor growth and activated immune response against melanoma-induced metastatic lung cancer [17]. Here, we found that NCI-H1299, which expresses high endogenous level of TLR3 protein (Figure 1B), did not succumb when exposed to increasing doses of polyI:C up to 50 μg/mL (Figure 1C), indicating insensitivity to polyI:C treatment. NCI-H1299 is probably saturated with TLR3 protein, which may explain for its unresponsiveness or insensitivity to polyI:C treatment. At the saturation level of TLR3, polyI:C may not effectively activate the TLR3-mediated apoptotic signaling, leading to a quiescent state as indicated by the downregulation of cleaved caspase 3 (Supplementary Figure 3C). Probably, NCI-H1299 expresses high but non-functional TLR3 protein that does not engage polyI:C. More importantly, our findings suggest that low-to-medium level of functional TLR3 protein expressed in A549, NCI-H292 and NCI-H358 appeared to support the susceptibility of these cells to polyI:C treatment. For example, A549 and NCI-H292 expressed low but adequate TLR3 protein (Figure 1B) for binding with polyI:C, resulting in suppressions of survival (Figure 1E), oncogenicity (Figure 2A, 2B) and metastasis (Figure 2C–2E). PolyI:C induces apoptosis of A549, NCI-H292, and NCI-H358 via direct activation of TLR3-caspase 3/8-dependent apoptosis pathway. Furthermore, TLR3 antibody-neutralization (Figure 3) and TLR3 siRNA knockdown (Figure 4) reversed the polyI:C-suppression of survival and metastasis of A549 and NCI-H292, suggesting that polyI:C specifically acts on TLR3 protein to exert anti-cancer functions. Consistent with the anti-cancer activity of polyI:C [45], our findings reveal how polyI:C alone exerts pro-apoptotic, anti-proliferative and anti-metastatic activities in susceptible lung cancer cells, to suppress survival and oncogenicity of A549, NCI-H292, and NCI-H358.

PolyI:C stimulation has been reported to activate inflammatory response through production of pro-inflammatory cytokines (IL-1β, IL-6, and IL-8) [47, 48]. Here, we showed that stimulation of different lung cancer cell lines with polyI:C induced differential secretion of inflammatory cytokines in a cell type-specific manner. Notably, NCI-H358, which expresses medium level of TLR3 protein and produces abundant endogenous IL6 and IL8, was not further induced by polyI:C to produce more of these cytokines (Figure 5). NCI-H358, which expresses high endogenous level of IL-6 protein, underwent IL6-independent suppression of metastasis when treated with polyI:C, and this was mediated indirectly through inactivation of IL6/JAK2/STAT3 signalling (Supplementary Figure 3C). Hence, NCI-H358 was unaffected by the inhibition of cytokine-dependent metastasis. On the other hand, NCI-H1299, which also expresses high endogenous level of TLR3, was insensitive/unresponsive to polyI:C stimulation, and did not secrete any pro-inflammatory cytokines (Figure 5). The apparent resistance/unresponsiveness of NCI-H1299 to polyI:C may be due to both the quiescence of TLR3 signalling pathway and the inactivation of IL6/JAK2/STAT3 signalling (Supplementary Figure 3C). Concordantly, A549 and NCI-H292 cells which express low but adequate levels of TLR3, were sensitive to polyI:C stimulation, producing high levels of pro-inflammatory cytokines (IL6, IL8 and GRO) associated with survival and metastasis (Figure 5C). IL6 was reported to stimulate STAT3 activity which promotes tumor growth and survival of NSCLC via JAK/STAT3 signalling [49]. Consistently, we found that inhibition of STAT3 by Stattic suppressed polyI:C-induced IL6 secretion in A549, indicating that polyI:C activates JAK2/STAT3 signalling to enhance the production of IL6 (Figure 6E). Thus, our findings suggest that polyI:C kills A549 via both activation of IL6/JAK2/STAT3 and TLR3-caspase-3/8 apoptosis pathways.

**Figure 9:**
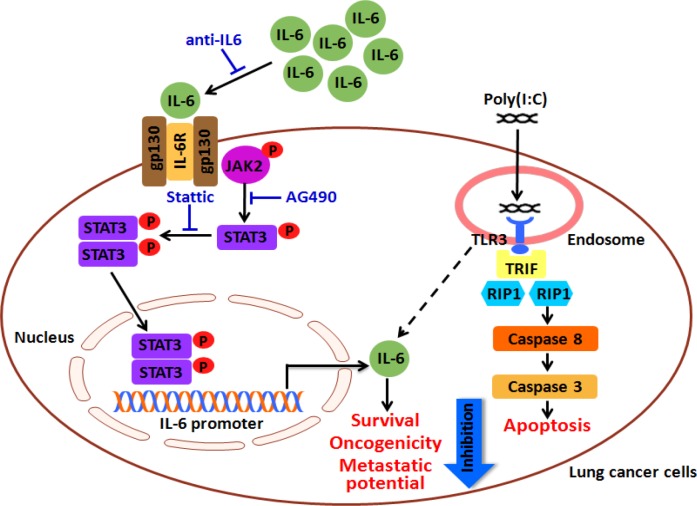
A hypothetical mechanism of polyI:C-suppression of survival, oncogenicity and metastatic potential of lung cancer cells Lung cancer cells which express low but adequate levels of TLR3 protein engages polyI:C, which in turn activates TLR3-mediated caspase 3/8 apoptosis pathway. Single treatment with polyI:C alone suppresses survival, oncogenicity and metastatic potential of lung cancer cells (e.g. A549, NCI-H292). In addition to apoptosis, polyI:C increases the secretion of IL6 through activation of STAT3 and JAK2. A combinatorial treatment with polyI:C and anti-IL6 antibody decreases IL6 production and subsequently enhances the polyI:C-killing and suppresses oncogenicity and metastatic potential of A549. Similarly, blockade of STAT3 and JAK2 activities by Stattic and AG490 antagonists, respectively, enhances the cancer killing effect of polyI:C. Taken together, a combinatorial treatment of polyI:C+anti-IL6 enhances the impact of polyI:C-killing of A549 through IL6/JAK2/STAT3 signalling pathway.

PolyI:C can be used as an anti-cancer therapy or a vaccine adjuvant. Combinatorial therapy with Hiltonol and siltuximab is known to control tumor growth and enhance local immune response, providing evidence that they not only attenuate survival and proliferation of cancer cells but also activate infiltration of immune cells [50]. Herein, we demonstrated that combinatorial treatment with polyI:C and anti-IL6 antibody enhanced polyI:C-mediated suppressions of survival, oncogenicity, and metastatic potential of A549 (Figure 7, Figure 8). Furthermore, blockade of the JAK2 and STAT3 activities enhanced the polyI:C-suppressions of survival, oncogenicity, and metastasis of A549 (Figure 7, Figure 8) and NCI-H292 (Supplementary Figure 4, Supplementary Figure 5). Our data suggest that enhancement of polyI:C-killing of A549 resulted from the blockade of IL6-dependent JAK2/STAT3 signalling, but polyI:C-killing of NCI-H292 resulted from the blockade of IL6-independent JAK2/STAT3 signalling. We postulate a model to illustrate this mechanism (Figure 9). It is conceivable that as long as a cancer cell (e.g. A549, NCI-H292, and NCI-H358) expresses a low-to-medium level of functional TLR3 protein, it will engage polyI:C and becomes responsive to polyI:C treatment, which activates the TLR3 signalling to subsequently kill the lung carcinoma. Thus, we propose that the expression of TLR3 and secretion of pro-/anti-inflammatory cytokines would correlate with the efficacy of polyI:C (and possibly, Hiltonol) treatment of lung cancer cells. Combination of polyI:C and anti-IL6 antibody enhanced polyI:C-suppressions of survival, oncogenicity, and metastasis of A549 and potentially other cancer cells, as long as they express sufficient but non-saturating levels of functional TLR3 protein. Blockade of IL6-dependent JAK2/STAT3 signalling enhanced polyI:C-killing of A549, alongside TLR3-caspase-3/8 apoptotic pathway. We have therefore uncovered the association of pro-inflammatory cytokine expression profiles with the TLR3-mediated apoptosis pathways, which could be utilised as indicators to predict therapeutic efficacy of lung cancers through their susceptibility to polyI:C treatment, with good prognosis. Our findings anticipate a promising outcome with the administration of Hiltonol (polyI:C-based therapy), either alone or in combination with siltuximab (anti-IL6 monoclonal antibody) and/or STAT3/ JAK2 antagonists, for treatment of lung cancers, particularly, the subtypes which express low-to-medium levels of TLR3 protein.

## MATERIALS AND METHODS

### Cell lines and reagents

Non-small cell lung cancer (A549, NCI-H292, NCI-H1299 and NCI-H358), hepatocellular cancer (SNU449, HuH1, HuH7, Chang, Hep3B, HepG2), colon cancer (WDr) and breast cancer (MCF-7) cell lines, obtained from American Type Culture Collection (ATCC), were cultured in complete RPMI 1640 medium (Gibco) supplemented with 10% FBS (Thermo Scientific) and 100 U/ml penicillin and 100 μg/ml streptomycin (Invitrogen). Polyinosinic-polycytidilic acid (polyI:C), is a synthetic short-chain analog of dsRNA from InvivoGen. Actinomycin D, 3-(4,5-dimethylthiazol-2-yl)-2,5-diphenyltetrazolium bromide (MTT), and Stattic were from Sigma. Tyrphostin AG 490 was from Calbiochem. Antibodies used were rabbit anti-IL6 polyclonal antibody (ab6672, Abcam), ChromPure rabbit and mouse IgG control, whole molecule (Jackson ImmunoReseach), functional grade purified anti-human TLR3 (e-Bioscience), mouse IgG1 (ICIG1) (FITC) isotype (ab91356, Abcam), anti-TLR3 antibody (40C1285.6) (FITC) (ab45053, Abcam), anti-STAT3 (phospho Y705) antibody and anti-STAT3 antibody (Abcam), anti-JAK2 and Phospho-JAK2 (Cell Signalling), Caspase-3 (8G10, Cell Signalling).

### PolyI:C stimulation

To investigate the therapeutic effect of polyI:C, lung cancer cells were treated with polyI:C as previously described [51, 52]. PolyI:C at increasing concentrations of 5-50 μg/mL in complete RPMI 1640 medium was added to the cells and incubated for 24 h at 37°C. The negative control was phosphate buffered saline (PBS) without polyI:C.

### Real time qPCR

Cells were harvested and lysed with Trizol reagent (Ambion) to extract total RNA according to the manufacturer's instructions. Total RNA of 2 μg was used for cDNA synthesis using Superscript III System (Invitrogen) with oligo(dT) primers. Real time qPCR was performed with GoTaq qPCR Master Mix (Promega) using a LightCycler 480 system (Roche) under the following conditions: 95°C (5 min), 45 cycles of 95°C (10 s) and 60°C (1 min). The expression of the housekeeping gene, beta-2-microglobulin (*B2m*) was used as an endogenous control. The qPCR primers used were: human *Tlr3* (F: 5′-TCTCATGTCCAACTCAATCCA-3′, R: 5′-TGGAGATTTTCCAGCTGAACC-3′), human *B2m* forward (F: 5′-TTCAGCAAGGACTGGTCTTTCTAT-3′, R: 5′-TGCGGCATCTTCAAACCTC-3′). *Hprt* (F: 5′-TGACACTGGCAAAACAATGCA-3′, R: 5′-GGTCCTTTTCACCAGCAAGCT-3′). The qPCR products were analysed in triplicates and the fold change of the gene expression was quantified relative to the housekeeping genes, *B2m* or *Hprt*, using the ΔΔCt method [53].

### Flow cytometry analysis

The expression of TLR3 protein in lung cancer cells was quantified by flow cytometry analysis. Cells were plated at 1×10^6^/well in 6-well plates and allowed to adhere overnight. Cells were harvested, washed in PBS, fixed and permeabilized using the Cytofix/Cytoperm Kit (BD Bioscience). Subsequently, the cell pellets were resuspended in Cytoperm buffer containing 2 μg FITC-conjugated anti-TLR3 antibody or FITC-conjugated mouse IgG1 isotype control (Abcam), for 30 min on ice in the dark, and washed twice. The cells were resuspended in Cytoperm buffer and analyzed on the BD LSRFortessa flow cytometer.

### Cell viability, apoptosis, proliferation, oncogenicity, cellular motility, migration and invasion assays

The following functional assays were performed to analyse the impact of polyI:C, anti-IL6 antibody and/or chemical inhibitors of JAK2 and/or STAT3, on the cellular behaviours of lung cancer cells. Where appropriate, we performed real-time imaging of the functional assays using the IncuCyte^®^ live cell analysis system, Essen BioScience Inc.

### Cell viability (MTT assay)

Cell viability was analysed by 3-(4,5-dimethylathiazol-2-yl)-2,5-diphenyl tetrazolium bromide (MTT) assay. Cells were plated in 96-well plates at 5×10^3^/well in complete RPMI 1640 medium and allowed to adhere overnight. The cells were treated with polyI:C at the indicated concentrations in medium containing 10% FBS, for 24 and 48 h followed by incubation with 10 μL of 12 mM MTT solution for 4 h at 37°C. The formation of formazan crystals in the viable cells were dissolved in 100 μL of SDS-HCl lysis solution (10% SDS in 0.01 M HCl) overnight at 37°C and the absorbance was read at 570 nm using spectrophotometer (BioTek).

### Apoptosis (caspase glo-3/7 assay)

Cells were seeded in 96-well white opaque plates at a density of 5×10^3^/well in 10% FBS-containing RPMI, and allowed to adhere overnight. The cells were treated with PBS (control) or 10 μg/mL polyI:C and incubated for 24 h. An equal volume of caspase Glo-3/7 reagent (Promega) was added and incubated for 30 min. Subsequently, apoptosis was determined by measuring caspase 3/7 luminescence activity using spectrophotometer (BioTek). Where appropriate, apoptosis was analysed by Caspase-3/7 apoptosis assay (Essen Bioscience) according to the manufacturer's instruction. Apoptosis was determined by fluorescence microscopy of the nuclei labeled with green fluorescent caspase-3/7. Labeled cells were visualized using an inverted phase-contrast fluorescence microscope (Carl Zeiss).

### Monolayer cell proliferation (total cell number assay)

The total cell number was assessed by monolayer cell proliferation. Overnight cultures of 5×10^4^ cells/well were treated with PBS (control) or 10 μg/mL of polyI:C over a period of 72 h. The proliferation of the cells was scored after each 24 h-incubation over three consecutive days. The cells were trypsinized with Trypsin-EDTA (0.05%) (ThermoFisher Scientific), and the viable cells were stained with trypan blue solution (0.4%, ThermoFisher Scientific) and enumerated using a hematocytometer.

### Oncogenecity

#### Soft agar colony formation assay

Anchorage-independent growth or anoikis of the lung cancer cells was assessed using the soft agar colony formation assay. Briefly, wells of a 96-well black plate were covered with a soft agar layer (0.5% agarose in serum free RPMI). A density of 5×10^3^ cells in 20% FBS RMPI medium were mixed with an equal volume of warm 0.7% agarose in serum free RPMI and 100 μL of the cell mixture was seeded on top of the 0.5% soft agar layer. Complete medium containing JAK2 inhibitor (AG490, Calbiochem) or STAT3 inhibitor (Stattic, Sigma) or anti-IL6 antibody (ab6672, Abcam), in combination with PBS or 10 μg/mL polyI:C was added to the cells and incubated at 37°C. The medium was replaced every 3 days until the experiment was terminated after 12 days. The colony formation of the cells were imaged and the cell viability was measured using Alamar blue (Invitrogen).

#### 3D matrigel growth assay

To examine the three-dimensional culture growth of cancer cells in matrigel, which represents a reconstituted basement membrane or extracellular matrix (ECM), a 96-well plate was coated with matrigel as a base layer. Cells at 5×10^3^/well in complete medium supplemented with 4% matrigel were seeded into the wells. Matrigel-containing (4%) medium with polyI:C alone or in combination with inhibitors or antibodies was replaced every 3 days until the experiment was terminated after 7 days. The 3D matrigel growth of the cells was imaged and the cell viability was measured using Alamar blue (Invitrogen).

### Metastatic potential

#### Cellular motility (scratch wound healing assay)

To examine the cell motility of the lung cancer cells under treatment, we performed scratch wound assay. Cells plated at a density of 5×10^5^/well in 90% confluency monolayer, were scratched with a sterile yellow pipette tip. The wounded cell monolayer was maintained in complete medium containing polyI:C alone or in combination with antibody or inhibitors until the wounds in one of the two compared groups were closed. The position of two frontlines of the cells migrating into the wound gap was monitored for 0, 12 and 24 h and cell images were captured at six fixed locations. The wound width was measured after scratching relative to the basal area as expressed in pixels, using ImageJ software. For IncuCyte live cell imaging of scratch wound healing assay, cells were cultured overnight at 37°C in a 96 well ImageLock™ Plates. Scratch wounds were made using a 96-pin auto-wound-making tool (WoundMaker; Essen BioScience). Subsequently, polyI:C (10 and 50 μg/mL) or PBS (control) was added and the migration of the cells was monitored over 24 h time course and images were captured at 30-min intervals by IncuCyte live-cell imaging system (Essen BioScience). The relative migration of the cells and wound width were calculated using IncuCyte analysis software.

#### Migration and invasion (transwell migration and invasion assays)

To examine the metastatic potential of cancer cells in response to a chemoattractant, we performed migration and invasion assays using 24-well cell culture inserts (8.0 μm membrane pores, BD Biosciences). For migration assay, cells (5.0×10^4^ A549, 1.0×10^5^ NCI-H292 or NCI-H358) in serum-free RPMI 1640 medium were seeded on the membrane of the transwell insert. As a chemoattractant, 10% FBS was used in the bottom chamber. For invasion assay, the cell culture inserts were coated with 2% matrigel with growth factor reduced (GFR) basement membrane matrix (BD Biosciences). Cells in serum-free medium were plated on the matrigel, and complete medium was then added into the bottom chamber of the companion plate. After 24 h-incubation at 37°C, cells were fixed with cold 4% paraformaldehyde and the upper surface of each membrane was removed with cotton swabs. The cells that had migrated to the underside of the membrane were stained with 4 μg/mL Hoechst 33258. The fluorescent nuclei were visualized with an inverted phase-contrast fluorescence microscope (Carl Zeiss), and the migrated cells were counted based on whole areas of transwell inserts.

### TLR3-gene specific RNA interference and antibody neutralization

To deplete the endogenous TLR3 expression, ON-TARGETplus SMARTpool human TLR3 siRNA (7098) and ON-TARGETplus Non-targeting control siRNA (scramble) were purchased from Dharmacon. The sequences for ON-TARGET plus SMARTpool human TLR3 siRNA were: 5′-GAACUAAAGAUCAUCGAUU-3′, 5′-CAGCAUCU GUCUUUAAUAA-3′, 5′-AGACCAAUCUCUCAAAU UU-3′, 5′-UCACGCAAUUGGAAGAUUA-3′ and ON-TARGETplus Non-targeting control siRNA were 5′-UGGUUUACAUGUCGACUAA-3′, 5′-UGGUUUAC AUGUUGUGUGA-3′, 5′-UGGUUUACAUGUUUUCU GA-3′, 5′-UGGUUUACAUGUUUUCCUA-3′. Transient transfection of 50 nM TLR3-siRNA into A549 and NCI-H292 lung cancer cells was performed using Lipofectamine RNAiMAX (Invitrogen) according to the manufacturer's protocol. After 24 h, the cells were incubated with PBS (control) or 10 μg/mL polyI:C for an additional 24 h before analysis. Blockade of TLR3 in the lung cancer cells by TLR3 antibody neutralization was performed as previously described, with slight modification [54]. Cells at a density of 5×10^4^/mL were plated in a 96-well plate and incubated with 50 ng/mL anti-TLR3 monoclonal antibody (functional grade purified, eBioscience) for 1 h at 37°C. The cells were then stimulated with 10 μg/mL polyI:C for 24 h before analysis.

### Functional antagonism assays with JAK2 and STAT3 inhibitors

For functional antagonism assays, lung cancer cells were treated with the following inhibitors: 200 μg/mL of anti-IL6 polyclonal antibody, 20 μM AG490 (JAK2 inhibitor) and 2 μM Stattic (STAT3 inhibitor) in combination with 10 μg/mL polyI:C to determine their inhibitory effects on cell viability and cellular behaviours. PBS alone without polyI:C was used as control.

### Cytokine arrays

The secretion of cytokines and matrix metalloproteinase from lung cancer cells induced by polyI:C was analysed using human cytokine antibody array C3 (AAH-CYT-3, RayBiotech) and human matrix metalloproteinase antibody array C1 (AAH-MMP-1, RayBiotech), respectively, according to the manufacturer's instructions. Cells were cultured in complete medium with polyI:C for 24 and 48 h. Culture supernatants were collected for cytokine antibody array analysis. Cytokine signal intensities were quantified using Image Studio Lite. The fold change of cytokines secreted from cells was calculated by normalization of polyI:C-treated samples to control PBS-treated samples. A heat map was generated by log using GENE-E software.

### ELISA quantification of cytokines

To quantify cytokines of interest: IL6, IL8, MCP-1, IL10, TNFα, IL-1β, IL-12p40 (based on the cytokine array results), the culture supernatants from the lung cancer cells treated with polyI:C for 24 and 48 h, were collected for the respective ELISA (BD Biosciences) according to manufacturer's instructions.

### Western blot analysis

Western blot analysis of specific proteins in the cell lysate and culture supernatant was performed and the intensity of the protein bands was read on ImageQuant LAS4000 (GE Healthcare). Western blot analysis was performed as previously described [55].

### Statistical analysis

All numerical data are expressed as means ± SEM from three independent experiments with biological replicates each. All treatment conditions and experiments were repeated at least three times. Statistical analysis was performed using GraphPad Prism 5.03 (GraphPad Software) or Excel 2013 (Microsoft). Data were analysed by two-tailed student's t-test and p value < 0.05 were considered significant.

## SUPPLEMENTARY MATERIALS FIGURES AND TABLES















## Abbreviations

FBS: fetal bovine serum
GRO: growth-regulated oncogene
HCC: hepatocellular carcinoma
IL6: interleukin 6
IL8: interleukin 8
MCP-1: Monocyte chemoattractant protein-1
MMP: matrix metalloproteinase
MTT: 3-(4,5-dimethylathiazol-2-yl)-2,5-diphenyl tetrazolium bromide
NSCLC: non-small cell lung carcinoma
PolyI:C: polyinosinic-polycytidylic acid
TLR3: toll-like receptor 3
