# Supplementary material for: Combinatorial treatment with polyI:C and anti-IL6 enhances apoptosis and suppresses metastasis of lung cancer cells
Source: Oncotarget. 2017 Mar 2;8(20):32884–904. doi: 10.18632/oncotarget.15862 (PMC5464836; doi:10.18632/oncotarget.15862)
Supplement: Supplementary file 1 [file oncotarget-08-32884-s001.pdf]

Combinatorial treatment with polyI:C and anti-IL6 enhances apoptosis and suppresses metastasis of lung cancer cells

SUPPLEMENTARY FIGURES

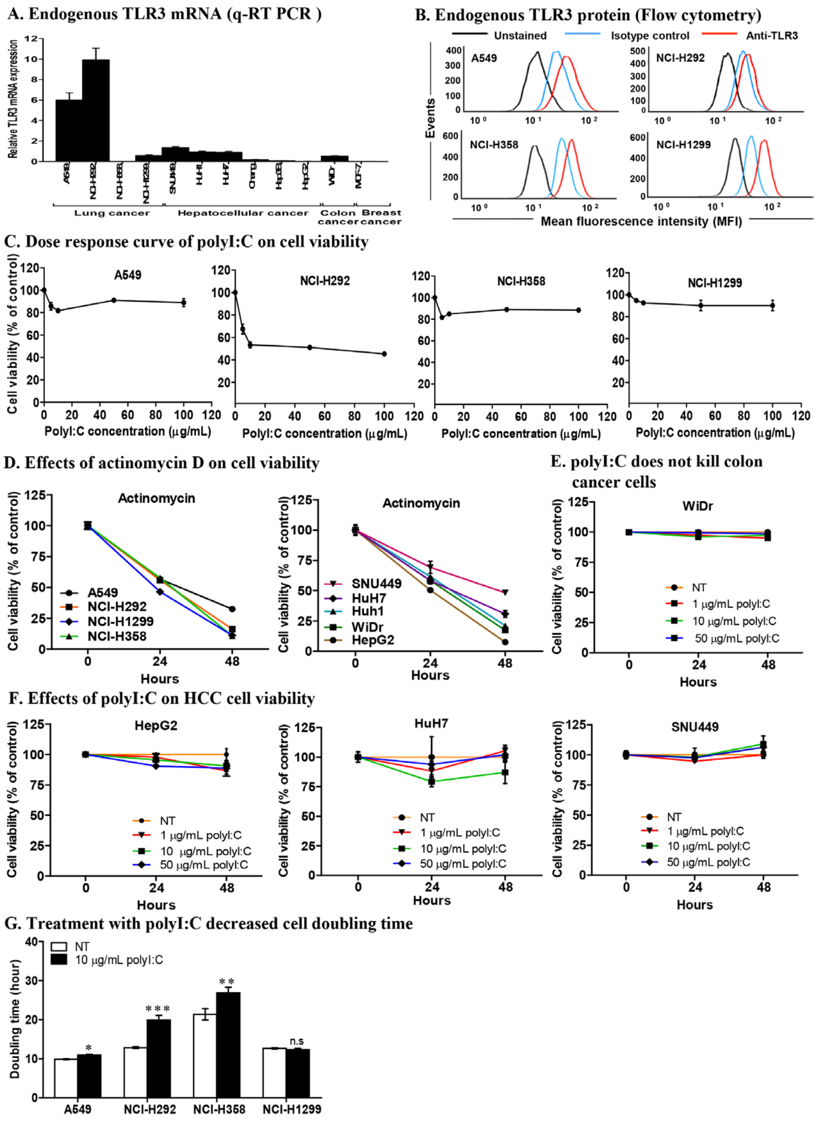

Supplementary Figure 1: Cancer cells display variable levels of endogenous TLR3 mRNA and differential susceptibility to polyI:C-killing. **(A)** Real-time qPCR analysis of endogenous *Tlr3* mRNA levels in lung, liver, colon, and breast cancer cell lines. Data are presented as relative *Tlr3* mRNA level compared to a housekeeping gene, *B2m*. **(B)** TLR3 protein in lung cancer cells was analysed by flow cytometry. Cells were intracellularly stained with FITC-conjugated IgG isotype control or FITC-conjugated anti-TLR3 antibody. **(C)** Dose response of polyI:C on cell viability of A549, NCI-H292, NCI-H358 and NCI-H1299. After 24-h treatment with different concentrations of polyI:C (1, 5, 10, 50, 100 μg/mL), the cell viability was measured by MTT assay. **(D)** Actinomycin D, a potent anti-neoplastic agent (used as a positive control), decreases cell viability of different cancer cell lines (lung, liver). Cells were treated with 1.0 μg/mL actinomycin D for 24 and 48 h and viability was analyzed by MTT assay. **(E)** Colon cancer cell line (WiDr) is resistant to polyI:C. **(F)** Cell viability of liver cancer cells (HepG2, HuH7, SNU499) treated with different concentrations of polyI:C. Cells were treated for 24 and 48 h with increasing concentrations of 1, 10, 50 μg/mL of polyI:C and viability was analyzed by MTT assay. NT (non-treated) indicates cells treated with PBS only. **(G)** Doubling time of lung cancer cells (A549, NCI-H292, NCI-H358, NCI-H1299) treated with 10 μg/mL polyI:C for 24 h. The total cell number was counted using improved Neubauer hemocytometer chamber. The doubling time was calculated by using online doubling time calculator (http://www.doubling-time.com/compute.php). *$P<0.05$; **$P<0.01$; ***$P<0.001$; n.s, non-significant.

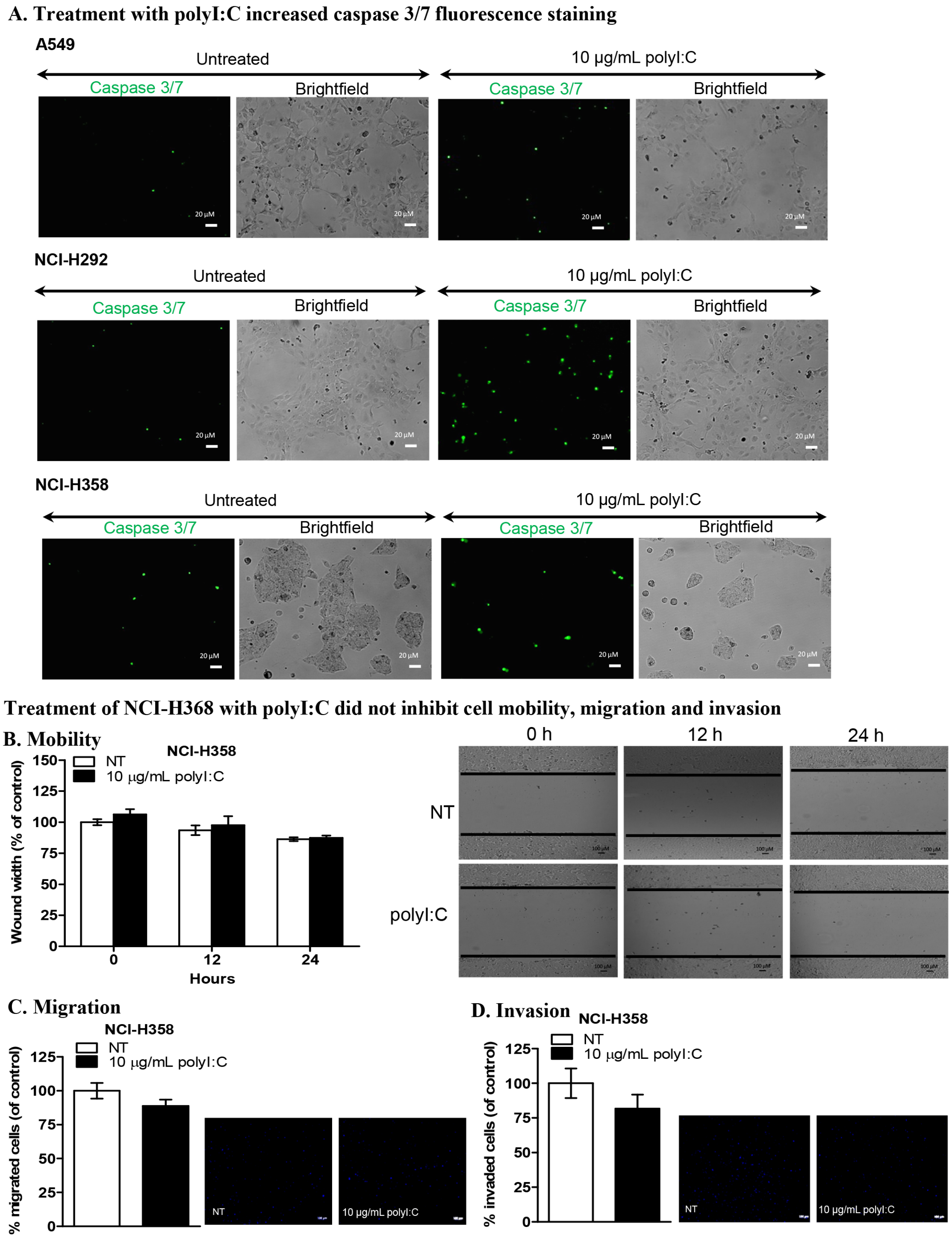

Supplementary Figure 2: PolyI:C induces caspase 3/7-mediated apoptosis in A549, NCI-H292 and NCI-H358, and suppresses cellular motility of A549 and NCI-H292. (A) Cells were treated with 10 μg/mL polyI:C for 24 h and stained with IncuCyte™ Caspase-3/7 apoptosis assay reagent diluted 1:5000 in complete medium +/- polyI:C. The morphological changes associated with apoptotic cells were observed under fluorescence microscopy. The nucleus of apoptotic cells labelled with green fluorescence indicates caspase 3/7-mediated apoptosis. Fluorescence and phase contrast images were taken at 100x magnification. Bar, 20 μM. **(B)** Cellular motility and wound closure rates of NCI-H358 treated with PBS (NT) or 10 μg/mL polyI:C, at time intervals of 0, 12, 24 h, showed slight and insignificant suppression over time. **(C)** Migration of NCI-H358 was determined by transwell migration assay. Cells were treated with PBS (NT) or 10 μg/mL polyI:C for 24 h and the migrated cells underneath the transwell insert were stained by Hoechst 33342, and counted under fluorescence microscopy. The representative images of migrated NCI-H358 cells were examined under 40x magnification. Bar, 100 μM. **(D)** Invasion of NCI-H358 was determined by 2% matrigel pre-coated transwell invasion assay. The same procedure was followed as described in **(C)**.

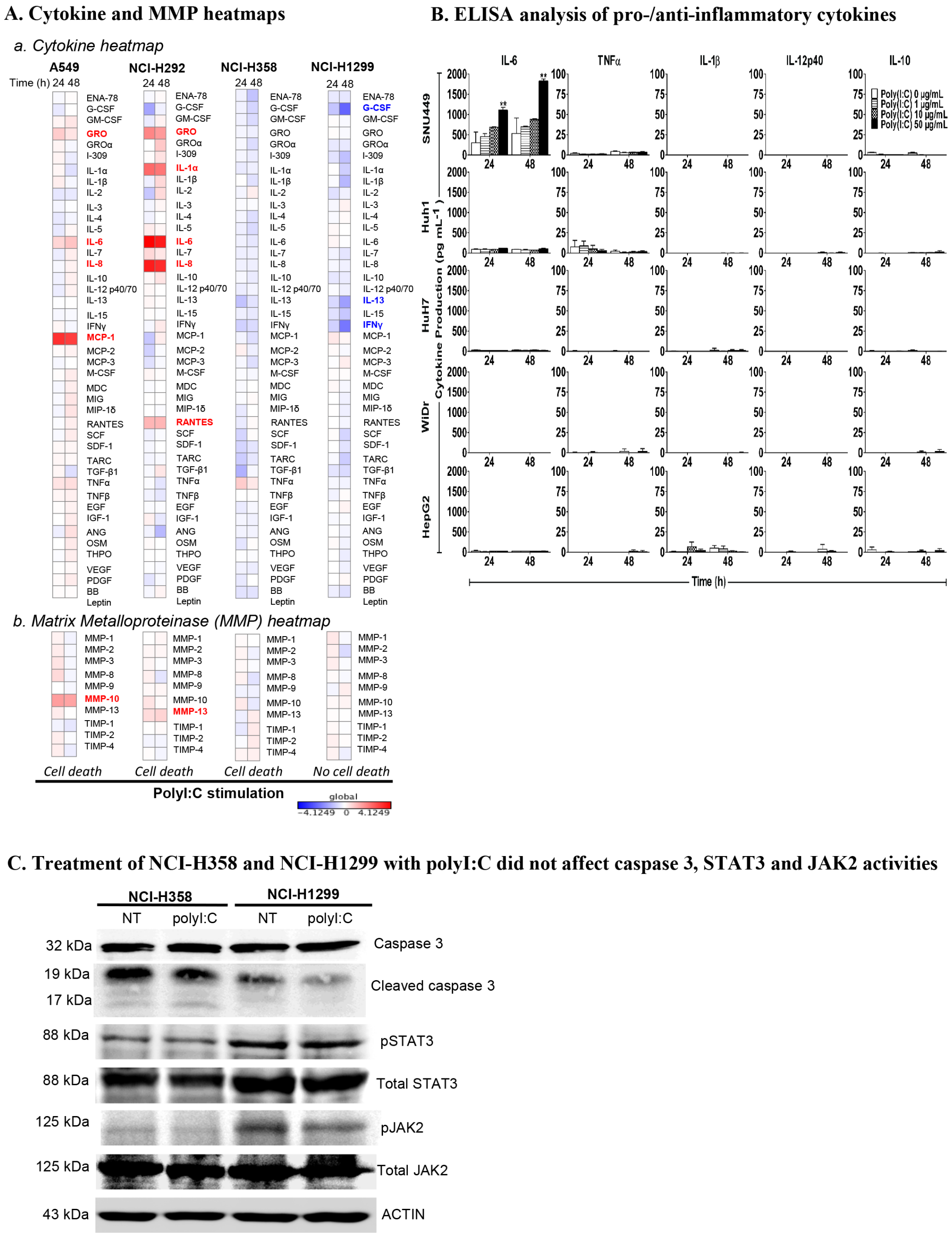

Supplementary Figure 3: Heatmap of the differential expression of cytokines and MMPs of lung cancer cells in response to 10 µg/mL polyI:C stimulation. (A) (a) PolyI:C induces differential cytokine production in lung cancer cells: A549 secreted GRO, IL6, IL8, MCP-1; NCI-H292 secreted GRO, IL-1α, IL-6, IL-8, RANTES; NCI-H358 produced none; NCI-H1299 secreted G-CSF, IL-13, INF-γ. (b) PolyI:C induces differential MMP expression in lung cancer cells: A549 (MMP-10); NCI-H292 (MMP-13); NCI-H358 and NCI-H1299 (none). PolyI:C-treated cells were normalized to control NT cells. Cytokines and MMP signal intensities were quantified using Image Studio Lite and the fold-change of polyI:C-treated cells was relative to NT. The log value of fold-change was used to generate the heat map. The fold-change of cytokine or MMP expression of the polyI:C-treated cells of >2-fold (highlighted in red) was considered as significant increase and <0.5-fold (highlighted in blue) was considered as significant decrease. **(B)** ELISA of secreted IL6, TNFα, IL-1β, IL-12p40, IL-10 in liver and colon cancer cell lines stimulated with 1, 10, 50 µg/mL of polyI:C for 24 and 48 h. PolyI:C increased IL-6 secretion in SNU449 cells in a dose-dependent manner but appeared to exert no significant changes in liver (Huh1, HuH7, HepG2) and colon (WiDr) cancer cells. **(C)** PolyI:C stimulation did not increase the expression of activated caspase 3 (19, 17 kDa) and phosphorylation of STAT3, and JAK2 in NCI-H358 and NCI-H1299. Cells were treated with 10 µg/mL polyI:C for 24 h and whole cell lysates were analysed by Western blotting. β-Actin was used as the loading control. ***P*<0.01.

A. Inhibitors of STAT3 (Stattic) and JAK2 (AG490) enhanced polyI:C-mediated apoptosis

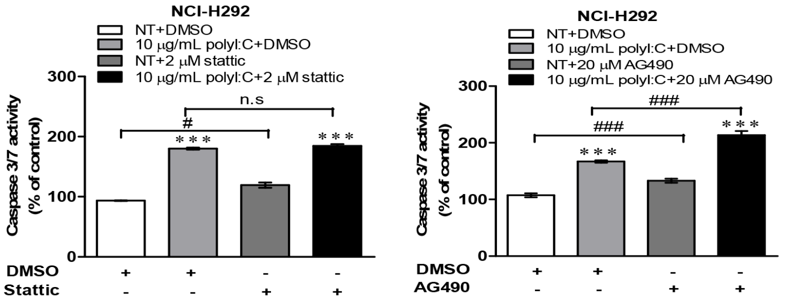

B. Inhibitors of STAT3 (Stattic) and JAK2 (AG490) enhanced polyI:C-mediated suppression of anchorage-independent growth

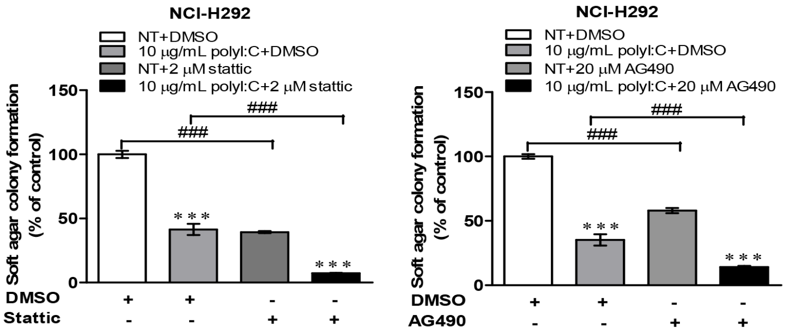

C. Inhibitors of STAT3 (Stattic) and JAK2 (AG490) enhanced polyI:C-mediated suppression of 3D matrigel growth

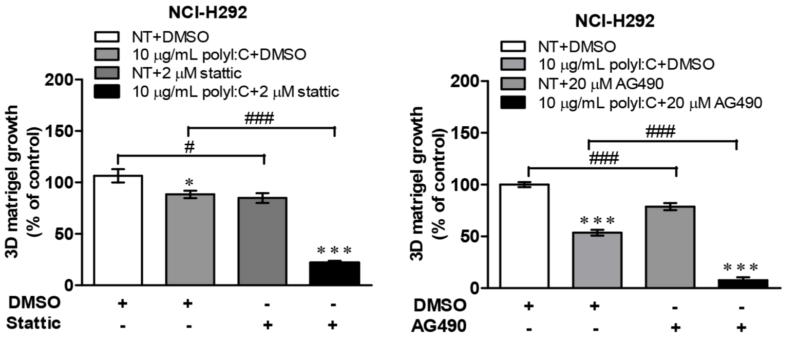

Supplementary Figure 4: Combinatorial treatment of NCI-H292 with polyI:C+Stattic or polyI:C+AG490 enhanced polyI:C-killing and further reduced oncogenicity. A combinatorial treatment for 24 h enhanced polyI:C-induced **(A)** apoptosis, **(B)** suppression of anchorage-independent growth and **(C)** suppression of 3D matrigel growth. Apoptosis is presented as percent of caspase 3/7 activity in the polyI:C-treated cells +/- a combinatorial treatment relative to NT. For anchorage-independent growth assay, cells were consecutively treated with a combinatorial treatment for 12 days and viability of the colony formed in the soft agar was analyzed by Alamar blue assay. For 3D matrigel assay, cells were consecutively treated for 7 days and viability of the colony formed in the matrigel was analyzed by Alamar blue assay. *P<0.05, **P<0.01, ***P<0.001 indicate polyI:C-treated cells vs. untreated cells. n.s, non-significant; $^{\#}$P<0.05, $^{\#\#}$P<0.01, $^{\#\#\#}$P<0.001 indicate cells treated with a combinatorial vs. no combinatorial treatment.

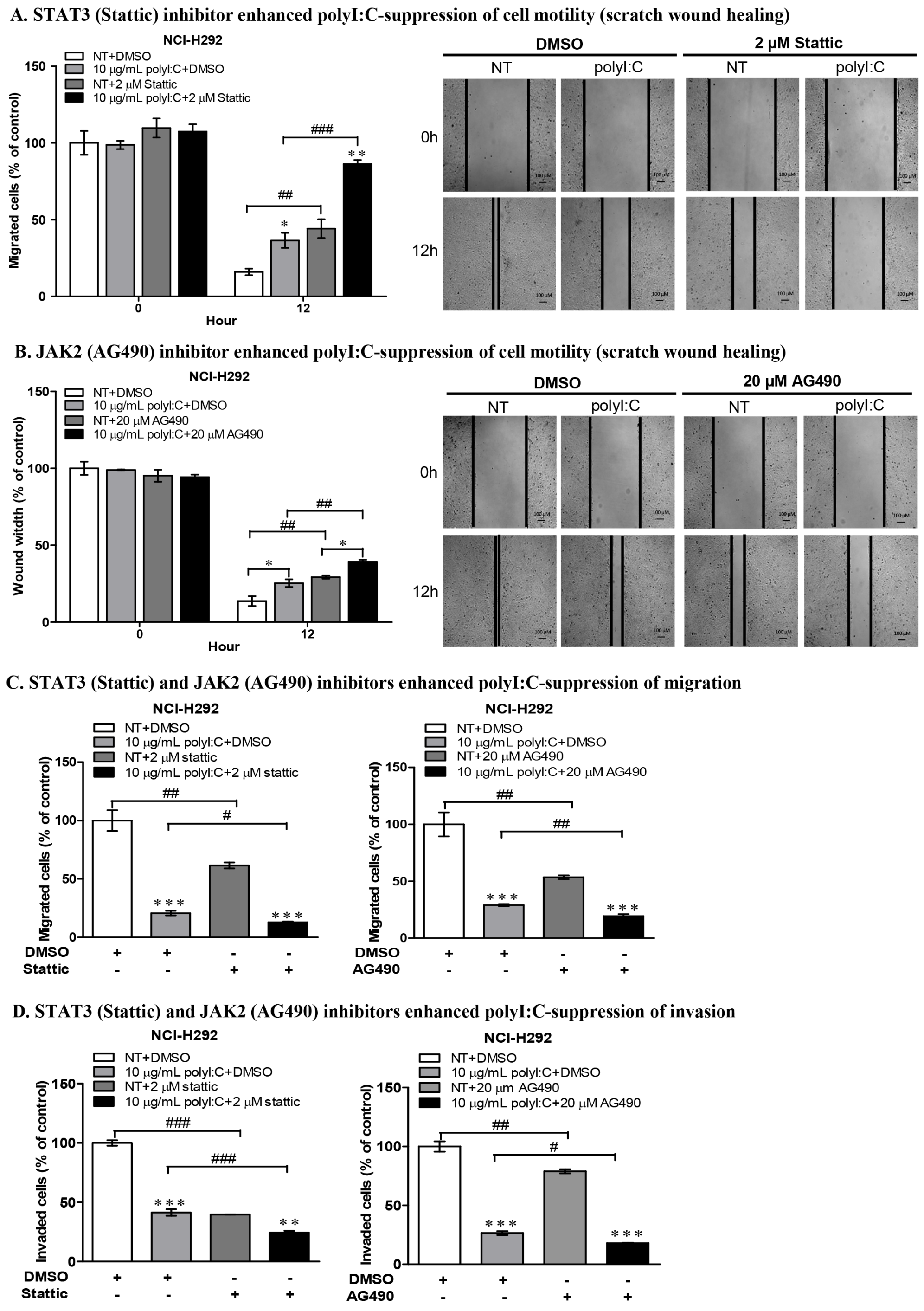

Supplementary Figure 5: Combinatorial treatment of NCI-H292 with polyI:C+STAT3 or polyI:C+JAK2 enhances polyI:C-suppression of cellular motility, migration and invasion. Cellular motility and wound closure rate of NCI-H292 treated with: **(A)** polyI:C+Stattic, **(B)** polyI:C+AG49, were measured by the scratch wound assay for time intervals of 0, 12, 24 h. Wound closure rates and wound width were measured using ImageJ software. The representative microscopy images were examined under 40x magnification. Wound width is presented as percent polyI:C treated cells +/- combinatorial treatment relative to NT cells at 0 h time point. **(C)** Migration and **(D)** Invasion of NCI-H292 treated with polyI:C+Stattic or polyI:C+AG490 for 24 h. The migrated or invading cells underneath the transwell insert were stained by Hoechst 33342 and counted under fluorescence microscopy. The representative microscopic images were examined under 40x magnification. Images were taken at 40x magnification. Bar, 100 μM; *P<0.05, **P<0.01, ***P<0.001 indicate polyI:C-treated cells vs. untreated cells. n.s, non-significant; #P<0.05, ##P<0.01, ###P<0.001 indicate cells treated with a combinatorial vs. no combinatorial treatment.

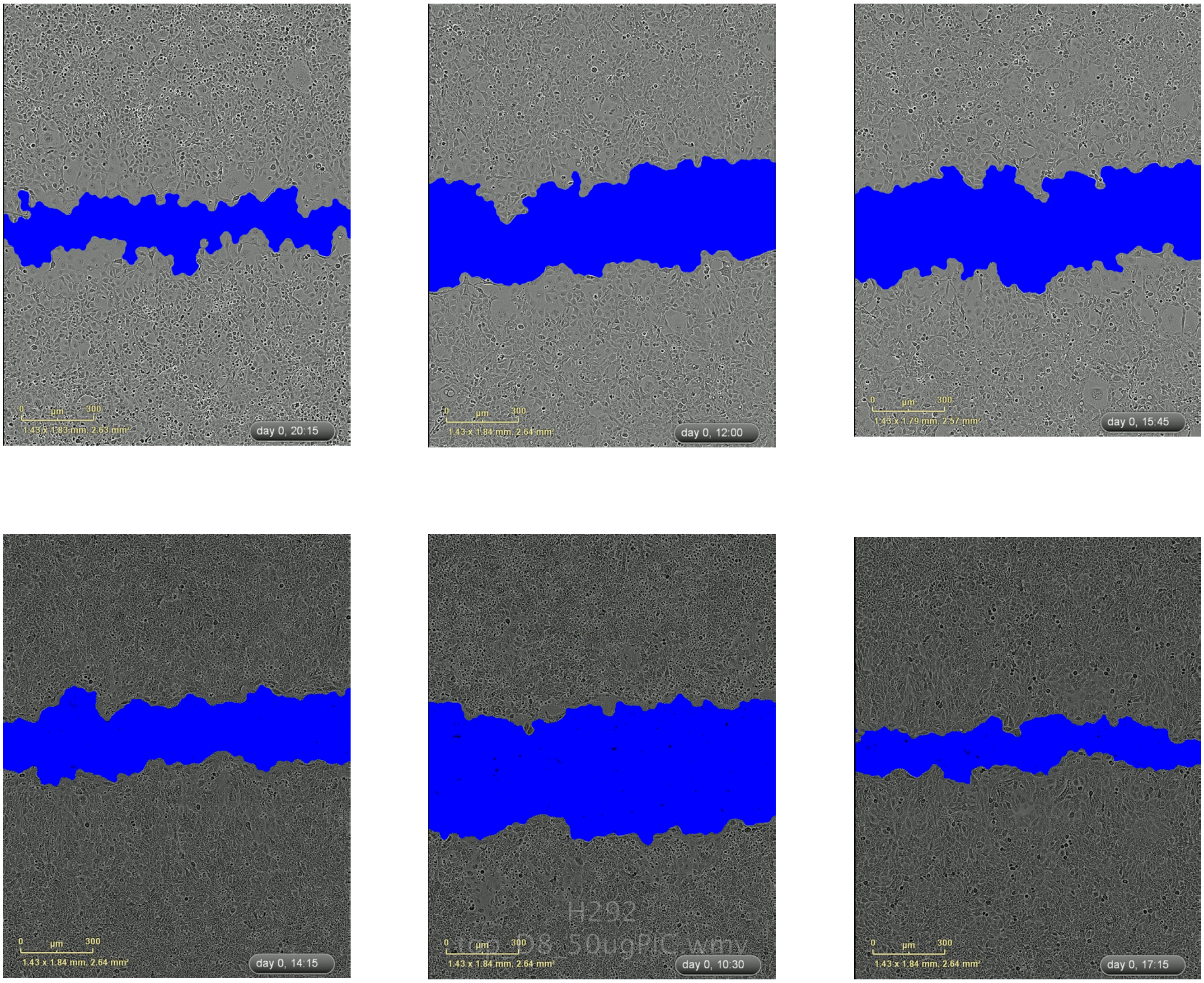

Supplementary Movie 1: PolyI:C suppressed cellular motility and wound closure rate of A549 and NCI-H292 as shown by time-lapse IncuCyte live cell imaging. Following scratch wounds using a 96-well wound-maker, cells were treated with different concentrations of polyI:C (10, 50 µg/mL) for 24 h. The cellular motility and wound closure rates were recorded by IncuCyte live cell imaging system. Time-lapse movies were created by recording images captured at 30-min intervals over 24 h. Representative movies are shown for the cellular motility and wound closure rates of the cells.
